# Disentangling structural and functional responses of native versus alien communities by canonical ordination analyses and variation partitioning with multiple matrices

**DOI:** 10.1038/s41598-022-16860-6

**Published:** 2022-07-27

**Authors:** Ioan Sîrbu, Ana-Maria Benedek, Bryan L. Brown, Monica Sîrbu

**Affiliations:** 1 Faculty of Sciences, Lucian Blaga University of Sibiu, 5-7 Dr. I. Raţiu St., 550012 Sibiu, Romania; 2 Department of Biological Sciences, Virginia Polytechnic Institute and State University, Blacksburg, VA 24060 USA; 3 Andrei Şaguna Pedagogical National College, 2 Turnu Roşu St., Hipodrom, Sibiu, Romania

**Keywords:** Ecology, Community ecology, Freshwater ecology, Invasive species, Environmental impact

## Abstract

Freshwaters are under accelerated human pressure, and mollusk communities are among its most sensitive, threatened, and valuable components. To the best of our knowledge, the overall effects of damming, environment, space, time, and invasive alien mollusk species, on structural and functional responses of native mollusk communities were not yet compared. Using historical information and recent data from a river, we aimed to investigate and disentangle these effects and evaluate the differences in structural and functional responses of natives and alien invasives to the same predictors. Variation partitioning showed that alien species were as important predictors as were environmental factors and time in explaining species composition of native freshwater mollusk communities. Aliens were more independent of environmental conditions than natives and responded to different drivers, partially explaining their invasion success. The increased abundance of some alien gastropods was positively related to taxonomic diversity, while certain alien bivalves were negatively associated with the functional diversity of native communities. We introduce a cumulative variation partitioning with multiple response (native and alien) and predictor matrices, along with a diagram to show their relations, advocating for a conceptual shift in future community ecology, from “variables to matrices” and from “multivariate analyses to multi-matrix statistical modeling”.

## Introduction

Freshwaters are at the extreme when ranking ecosystems by a multicriterial system, including, but not limited to, their use, functions, requirements, abundance, availability, and threats. They are at the forefront of the global biodiversity crisis^1^ as the least abundant, most limiting, necessary for human well-being, most threatened, and least effectively protected habitats^2^. The causes of imperilment are many, complex, and interacting^3,4^. Global human impacts have reduced the potential value of freshwater ecosystem services^5^, ranging from more than one-third (for commodities, biodiversity, and water quality) to about one-tenth (for water quality and greenhouse gasses). Termed "inland islands," freshwaters are prone to changes when altered or disturbed. They are particularly susceptible to alien invasive communities, which define the dawn of the "Exocene"^6^, a new epochal phase, the globally alien-dominated "bio-historical horizon." However, qualifying the effects of alien species is not a straightforward assertion. For instance, meta-analysis based on more than 200 publications aiming at characterizing relationships between native and alien species in all environments based on their traits and relationships to the environment revealed highly equivocal results regarding the actual impacts of invasive species^7^. Another review revealed that costs related to damage and economic losses are mostly miscalculated, while their benefits are usually neglected^8^.

Hydrologic alterations such as damming and reservoir building interact with alien invasive species (AIS) and enhance invasion risk^9^, but their relationships to environmental changes and effects on native communities are still little known and understudied despite their enormous conceptual and practical importance^10^. Most evidence suggests that damming of rivers causes functional structure shifts in fish communities^11^, simplification of their functional diversity^12^, loss of yield in fish stocks^13^, diversity reduction and homogenization of phytoplankton^14^, decreased biotic water quality indices and metrics in macroinvertebrate communities^15^, shifts in riparian vegetation^16^, impacting mollusk communities^17–21^, and others.

Aquatic invertebrate abundance and richness generally decrease below dams with the highest impacts on insect taxa, resulting in shorter food chains, simplification of food webs, and other effects^22^. In contrast, novel evidence suggests that anthropogenic habitats (ranging from small ponds to large reservoirs and canals), if properly managed, may provide undervalued prospects for the conservation of threatened species, for instance, freshwater mussels^23^.

Mollusks (gastropods and bivalves) are a crucial component of aquatic ecosystems, providing many services^24^ and are exceptionally threatened with extinction^1^ by habitat alteration, biotic interactions, and global change^25–27^. Despite their importance and precarious conservation status, knowledge of freshwater mollusks is limited^28^. The ''within enemy'' effect—when globally invasive species pose threats to related native communities—can affect the native mollusk communities, having a negative impact on ecosystem functions, human society, and its economy. Bivalves such as the Chinese giant mussel (*Sinanodonta woodiana*), Asian clam (*Corbicula fluminea*), and Zebra mussel (*Dreissena polymorpha*) have been shown to have devastating effects on the ecosystems they invade. Their features, traits, and effects as invaders have been documented in many studies^29–33^. In contrast, the effects of alien gastropods are far less studied. Despite extensive research, there is no agreement on how, why, and to what extent invasive mollusk species impact ecological and economic systems.

The overall effects of hydro-technical works like dams and reservoirs on native freshwater mollusk communities require further elucidation, particularly their interactions with local environmental factors, spatial structuring factors, mollusk invasive species, and functional traits. This combination of drivers has not been studied synthetically to the best of our knowledge. Therefore, we used a single analytical framework to investigate the effects of hydro-technical works on native mollusk communities by simultaneously evaluating their interaction with invasive mollusks, local environment, and spatial factors.

We aimed to: (1) restore and use historical information (from collections and old publications) as valuable sources for ecological statistical models; (2) disentangle and test the effects of hydro-technical works—especially building of reservoirs (dams for hydro energetic power)—environment, space, time, and mollusk AIS on the structural and functional dynamics of native freshwater mollusk communities; (3) investigate the differences in responses of natives and AIS to the same predictors, and characterize the reversed effects—the ability of communities to predict external variables, (4) test the effects of alien species on the structural and functional diversity of natives, and (5) develop a novel approach and method for analyzing and expressing relationships between native and alien communities while accounting also for their responses to environment and space. Analysis of differences in responses of native and AIS to predictor matrices, while accounting also for the residual (unexplained) variation, might bring new insight into the relationships between the heterogeneity of external factors characterizing the riverscapes and the communities adapted to these.

## Methods

### Research area

The study area is an 83 km section along the middle Olt River, between the town of Făgăraș (45.8512° N, 24.9733° E) and the Carpathian gorges (45.5317° N, 24.2721° E), in the region of Transylvania, central Romania. This reach was chosen primarily for the availability of historical and new data, representing periods before and after the major human impact of constructing six reservoirs during the second half of the XXth century.

### Mollusk communities' ecological parameters

We used literature and historical collections to gather all the available data on freshwater mollusk species from our target stream reach. There are reliable data about the freshwater mollusks from the XIXth century, from Bielz^34^, Kimakowicz^35^, and other naturalists of the Transylvanian Society for Nature Sciences in Sibiu, whose collections are kept mainly in the Natural History Museum of the Brukenthal National Museum in Sibiu, Romania. The historical data were subject to an exhaustive revision of the collections and literature^36^, and they represent the baseline before river alteration. This period was coded T1, as we considered time as factorial (denoted Period). The data from the post-impact period, T2, after the building of reservoirs and river damming, was collected between 1995 and 2000 and was derived from original research^37^ and literature^38^. To establish current conditions, during May and July 2020 (denoted T3), we surveyed the present-day mollusk community structure. Presence-absence of freshwater mollusk species from these periods in 11 consecutive sectors of the Olt River was related to matrices of environmental variables and species functional traits. But the original T3 data are counts in 20 sampling stations, used also in a second set of analyses. Sampling stations were selected to cover the whole research area and reflect historical data position. They are located upstream, downstream, and between the dams and reservoirs. The sectors and stations are placed in the same reach of the river; historical data were placed in sectors because they could not be ascribed to a specific location.

In 2020, we performed in each station three dredgings several meters apart. We washed the material, counted big snails and bivalves releasing them on the site, and preserved the remainder in alcohol for further study in the laboratory. We identified all mollusks to species level. In figures, species are referred to by the first three letters of the genus and species names (Table 1). For the native component of the freshwater mollusk community, we computed taxonomic diversity measures: species number (SNat), Hill's (N2) diversity, Shannon (H) entropy, and Pielou's evenness index (J), which relates H to the logarithm of species number^39^. We assessed the functional diversity (FD) of native communities using the Rao quadratic entropy^39,40^, separately for natives—FD(Natives), and AIS—FD(AIS). For T1–T3 data, we also assessed the Rao functional diversity for the whole community (native and AIS) —FD(Rao). Because the functional traits are a mixture of factorial and quantitative variables, we performed principal coordinates analysis (PCoA) on all traits using the Gower distance, saved the axes scores on all axes with non-zero eigenvalues, and calculated the FD on these scores and the corresponding community (sites-by-species) data tables.

**Table 1:** List of freshwater mollusk species identified in the study area, related to time and origin.

Species	Common name	Code	T1	T2	T3	Origin
*Theodoxus transversalis* (C. Pfeiffer, 1828)	Striped nerite	Thetra	1	0	0	NAT
*Viviparus acerosus* (Bourguignat, 1862)	Danube river snail	Vivace	0	0	1	AIS
*Bithynia tentaculata* (Linnaeus, 1758)	Faucet snail	Bitten	0	0	1	NAT
*Lithoglyphus naticoides* (C. Pfeiffer, 1828)	Gravel snail	Litnat	1	0	0	NAT
*Valvata piscinalis* (O.F. Müller, 1774)	European valve snail	Valpis	0	1	1	NAT
*Galba truncatula* (O.F. Müller, 1774)	Dwarf pond snail	Galtru	0	1	1	NAT
*Stagnicola corvus* (Gmelin, 1791)	Giant pond snail	Stacor	0	0	1	NAT
*Radix auricularia* (Linnaeus, 1758)	European ear snail	Radaur	0	1	1	NAT
*Lymnaea stagnalis* (Linnaeus, 1758)	Great pond snail	Lymsta	0	1	1	NAT
*Physa acuta* (Draparnaud, 1805)	Accute bladder snail	Phyacu	0	1	1	AIS
*Planorbarius corneus* (Linnaeus, 1758)	Great ramshorn	Placor	0	1	1	NAT
*Planorbis planorbis* (Linnaeus, 1758)	Common ramshorn	Plapla	0	1	1	NAT
*Bathyomphalus contortus* (Linnaeus, 1758)	Twisted ramshorn	Batcon	0	0	1	NAT
*Gyraulus albus* (O.F. Müller, 1774)	White ramshorn	Gyralb	0	1	1	NAT
*Gyraulus crista* (Linnaeus, 1758)	Nautilus ramshorn	Armcris	0	0	1	NAT
*Ferrissia californica* (Rowell, 1863)	Fragile Ancylide	Fercal	0	0	1	AIS
*Ancylus fluviatilis* O.F. Müller, 1774	Common river limpet	Ancflu	1	1	1	NAT
*Unio pictorum* (Linnaeus, 1758)	Painter's mussel	Unipic	0	0	1	NAT
*Unio crassus* Philipsson, 1788	Thick shelled river mussel	Unicra	1	0	1	NAT
*Anodonta cygnea* (Linnaeus, 1758)	Swan mussel	Anocyg	0	1	0	NAT
*Anodonta anatina* (Linnaeus, 1758)	Duck mussel	Anoana	1	0	0	NAT
*Sinanodonta woodiana* (Lea, 1834)	Chinese pond mussel	Sinwoo	0	0	1	AIS
*Corbicula fluminea* (O. F. Müller, 1774)	Asian (basket) clam	Corflu	0	0	1	AIS
*Musculium lacustre* (O. F. Müller, 1774)	Capped orb mussel	Muslac	0	1	1	NAT
*Pisidium casertanum* (Poli, 1791)	Ubiquitous peaclam	Piscas	1	1	1	NAT
*Pisidium nitidum* Jenyns, 1832	Shining peaclam	Pisnit	0	0	1	NAT
*Pisidium milium* Held, 1836	Quadrangular pillclam	Pismil	1	0	0	NAT
*Dreissena polymorpha* (Pallas, 1771)	Zebra mussel	Drepol	0	0	1	AIS

Code—code of species; Period: T1—XIXth century, T2—1995–2000, T3—2020; Origin: NAT—native species; AIS—alien invasive species; Occurrence: 1—presence, 0—absence.

The predictors used for T1–T3 binary data were: Period (time), Hab (habitat category) with levels (R) river, (L) lakes (i.e., reservoirs) and (C) canals, and Impact (human pressure), an ordinal variable ranging from 0 (natural conditions in the late 1800s) to 7 (most apparent impact on a river, meaning stagnant waters, places near dams, between concrete dykes). For T1, predictors were set to baseline (Hab = R and Impact = 0, meaning river close to natural status).

For the T3 count data, the corresponding predictors included the formerly described ones and, in addition, the Flow, an ordinal assessment of the average flowing conditions of the waters, ranging from 1 (almost) stagnant to 5, permanent, rapid, turbulent flow, and the distance to the nearest dam downstream, Dis_dam, in m. Data analysis was performed separately on the whole community data set and on AIS in relation to the native communities.

We used eleven functional traits (FT), but only some were selected during the forward selection procedures within the double-constrained ordination method and thus included in the analyses. The traits are: Taxon—factorial variable, with the levels (PG) prosobranch and (G) pulmonate gastropods, (U) Unionoida and (V) Veneroida bivalves; Origin—factor with levels (NAT) native and (AIS) alien invasive species; Habitat preferences—factor with (Rheo) rheophilic, (Lent) lentiphilic, (Phyt) phytophilic, (Ubi) ubiquitous; SizeM, maximal shell size—ordinal variable with the values: (1) < 2.5 mm, (2) 2.6–5 mm, (3) 5.1–15 mm, (4) 15.1–50 mm, (5) 50.1–100 mm, (6) > 100 mm; Sexes—factor with (S) separate sexes and (H) hermaphrodite; Ovipos, oviposition—factor with (OV) ovo-viviparity, (CAP) capsule/eggmass, (BE) parental care, juveniles in brood pouches of demibranchs, (No) no oviposition (external fecundation); Nooff—number of offspring, eggs or live young, ordinal variable with (1) 1–10, (2) 11–100, (3) 101–1000, (4) > 1000; Sexmat, sexual maturity—ordinal variable with (1) < 1 year, (2) about 1 year, (3) > 1 year; FeedT, feeding type—factor with (SCR) scraper, (SS) scraper and sediment, (SF) scraper and filter, (F) filter, (SEDF) suspension and deposit feeder; Food, food type—factor with (DAP) detritus, algae, higher plants, (CDA) detritus, algae, plants, carnivorous, (MAB) organic matter, bacteria, algae, detritus; and Light preferences (or tolerance)—ordinal variable with (1) mainly deep shade, (2) mainly light shade, (3) no shade. In the double-constrained correspondence analyses (dc-CA), we excluded some traits (Taxon, Origin, Habitat) to avoid circular reasoning. The values for the traits were based on the works of^41–45^.

### Data analysis

We used dc-CA with the forward selection of predictors and dimensionality testing^39,46^, redundancy analysis (RDA) and canonical correspondence analysis (CCA), and their attribute graphs (t-value biplots), variation partitioning with simple and conditional effects tested, species response curves using generalized linear models (GLM) or loess functions, and contour plots. To plot and test the Rao functional diversity measure^39,40^ in ordination space, we used generalized additive models (GAM). For model assessment and validation, we used adjusted explained variation and *p*-values adjusted by false discovery rate (FDR) to control the inflation of Type I error in multiple testing. We used the Monte Carlo test with 999 permutations for model evaluation, with a hierarchical permutation scheme for T1–T3 data and an unrestricted permutation scheme for T3 models. In the hierarchical permutation scheme, whole plots were sectors, considered linear transects, and split plots were the three time periods, considered time series. The analyses were performed in Canoco 5.15^39,40^. We present the methods in detail in the Supplementary Information for each analysis and illustration. We used the chi-squared (χ^2^) test for comparing classes of explained variance.

We performed a special set of analyses and introduced an algorithm for the complete Cumulative Variation Partitioning with Multiple Response and Predictor Matrices and its graphical representation (CUVARP method and diagram). The algorithm for these analyses comprised the following steps: (1) The native and alien communities (sites-by-species), and the environment matrices were each subjected to PCoA using the Gower distance. For the space matrix (spatial coordinates), we used the distance-based Moran Eigenvector Maps (db-MEM), using the nearest neighbor and the euclidean distance (we kept in the analyses axes with positive eigenvalues). The PCoA axes scores were saved in four tables, denoted as H-all (natives), A-all (aliens), E-all (environment), and S-all (space coordinates). (2) We used, in turn, H-all and A-all as response data tables, with the other three tables of PCoA scores as matrices of explanatory variables, in variation partitioning analyses, using RDA (with and without covariates). By reaching a consensus between these analyses through interactive forward selection of predictors, we selected the axes scores significantly related to each other, these being termed H (natives, scores of PCoA axes 1 and 2), A (aliens, scores of PCoA axes 1 and 5), E (environment, scores of PCoA axes 1 and 6), and S (space, scores of PCoA axes 1 and 3). (3) We assessed the variability of each table by a Principal Components Analysis (PCA) and calculated the total variation in the data by adding these values. (4) Each of the four data tables with selected axes scores (H, A, E, and S) was used as a response matrix, the other three being predictors, accounting for a complete disentangling of simple and conditional effects and evaluation of residual variation, repeatedly using RDA. (5) We built models as a table, and graphs (the CUVARP diagrams) depicting all parts resulted from variation partitioning, expressed as explained variation (sum of values obtained in the former steps) and percents. The whole algorithm is explained in the Supplementary Information, where we also present an overview of its shortcomings, alternatives, and some methods for expanding this framework. The variation partitioning illustrations were constructed using functions in "eulerr"^47^ and "nVennR"^48^ R packages.

### Ethics declarations

All aspects of fieldwork and animal handling complied with EU Council Directive 86/609/EEC on the experimental use of animals. Because the research area is part of the ROSCI0132 Middle Olt-Cibin-Hârtibaciu protected site, sampling was done with the approval of the Sibiu subsidiary of the National Agency for Protected Natural Areas (approval 430/ST SB/27.07.2020) Subsequently two working protocols were also approved by the Ethical Commission for Biomedical Research (approval 10/20.07.2021) and the newly established Ethical Commission for Scientific Research (approval 1/20.06.2022) of the Lucian Blaga University of Sibiu.

## Results

### Time dynamics of the mollusk communities

In this section, the presence-absence of the species recorded in the three periods (T1, T2, T3) are analyzed in relation to time, habitat, and human impact. The list of the 28 species of freshwater mollusks (17 gastropods and 11 bivalves) in T1–T3, their codes, and origins are given in Table 1.

The number of mollusk species has increased in time as the river has shifted from lotic to a mixture of flowing and stagnant sectors due to the building of reservoirs. T1 was characterized mainly by rheophilic elements and prosobranchs. Some species became extinct during the hydro-technical works (before or during T2) and are unlikely to recover, such as the rheophilic *Theodoxus transversalis* and *Lithoglyphus naticoides*. Other rheophilic species disappeared between T1 and T2 but managed to survive in tributaries and repopulated some sectors during the last years. The most remarkable recovery is that of the thick-shelled river mussel *Unio crassus*, a species protected by EU legislation. T2 was characterized by some extinctions but also colonization by lentiphilic pulmonates and tolerant, resistant species such as some clams. A few lotic species also survived in the river sectors between the dams. In T3, we encountered a rich and diverse community, including some newly established populations of AIS and the discontinuous presence of both lentic and lotic communities. Overall, the present-day fauna is richer than in former periods, consisting of 15 species of gastropods and 8 bivalves. The AIS included the gastropods *Physa acuta* and *Ferrissia californica*, which arrived in the area most likely during the XX^th^ century, *Viviparus acerosus*, which is native to the Danube, but unknown until after 2000 in the upper-middle Olt River basin, the bivalves *Dreissena polymorpha*, also native in the Danube but an invader in the middle Olt since 2008–2010, *Sinanodonta woodiana*, first found in 2015, and *Corbicula fluminea*, which was first discovered in the Olt (and also in Transylvania) during our survey in February 2020. The mean number of native species per river's sector increases almost linearly (2.8 species per sector in T1, 3.3 in T2, and 4.6 in T3), while the corresponding values for AIS increase non-linearly (no AIS in T1, 0.6 species per sector in T2 and 3.2 in T3).

**Figure 1:**
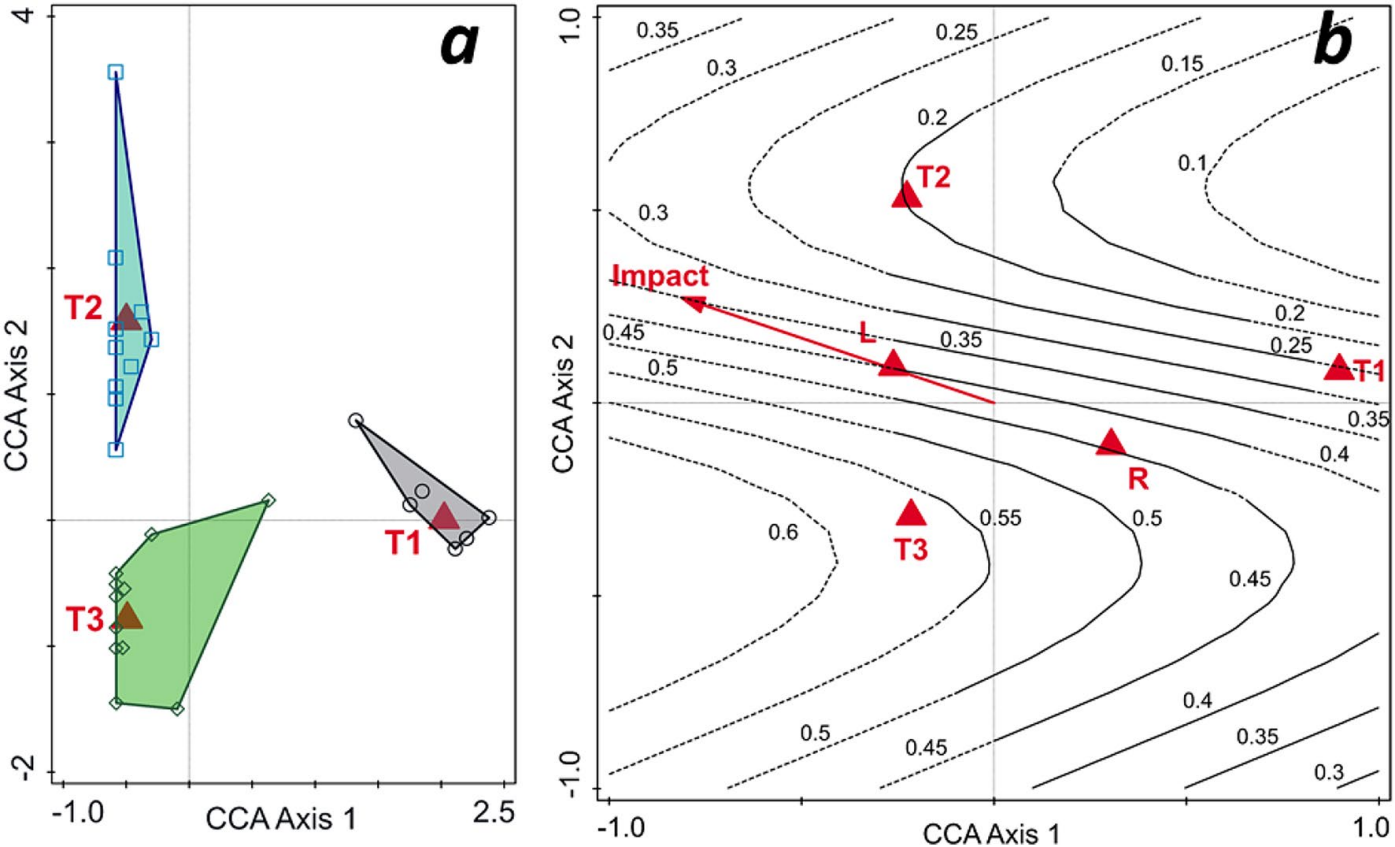
Canonical correspondence analysis (CCA) of mollusk communities: (**a**) classification diagram of sampling sites based on period (as predictors): T1—XIXth century, T2—1995–2000, T3—2020 (adjusted explained variation 23.6%; first axis accounts for 17.6% the second for 6.0%, both axes are significant, *p* = 0.001); (**b**) CCA diagram of species occurrence constrained by environmental predictors (period, habitat: L—lakes, lentic sector in reservoirs, R—river, lotic sectors, and Impact—human impact) with functional diversity expressed as Rao quadratic entropy index (FD (Rao)) isolines plotted by generalized additive models (GAM) on the ordination space (adjusted explained variation 28.36%; first axis accounts for 16.3%, the second for 6.0%, both axes are significant, *p* = 0.001) .

In the CCA of freshwater mollusk community changes through time (Period as predictor), the adjusted explained variation was 23.6% (test on all axes, pseudo-*F* = 5.9, *p* = 0.001). The polygons delimiting the positions of the sites during the three periods of time show no overlap, and they were distinct and separated in the ordination space (Fig. 1a). T2 (the period with maximum human impact) is distinctly placed and separated from the period without impact (T1) along both ordination axes. Meantime, T3 is closer to T1, having an intermediate position between the other two periods, showing a trend of recovery, such as the return of some species. In the CCA of T1–T3 species presence-absence predicted by the selected environmental descriptors (Period, Habitat, and Impact) (Fig. 1b), the adjusted explained variation was 28.36% (test on all axes, pseudo-*F* = 4.2, *p* = 0.001). FD(Rao) computed on all FT was plotted as isolines by GAM on the ordination space (model AIC = -17.19, model test *F* = 5.1, *p* = 0.003; tests of non-linearity in predictor effects: *F* = 3.9, *p* = 0.03). The functional diversity decreased from T1 to T2, then increased sharply to T3; it also decreased from rivers (R) to lakes (L) and along the human impact gradient (Impact).

**Figure 2:**
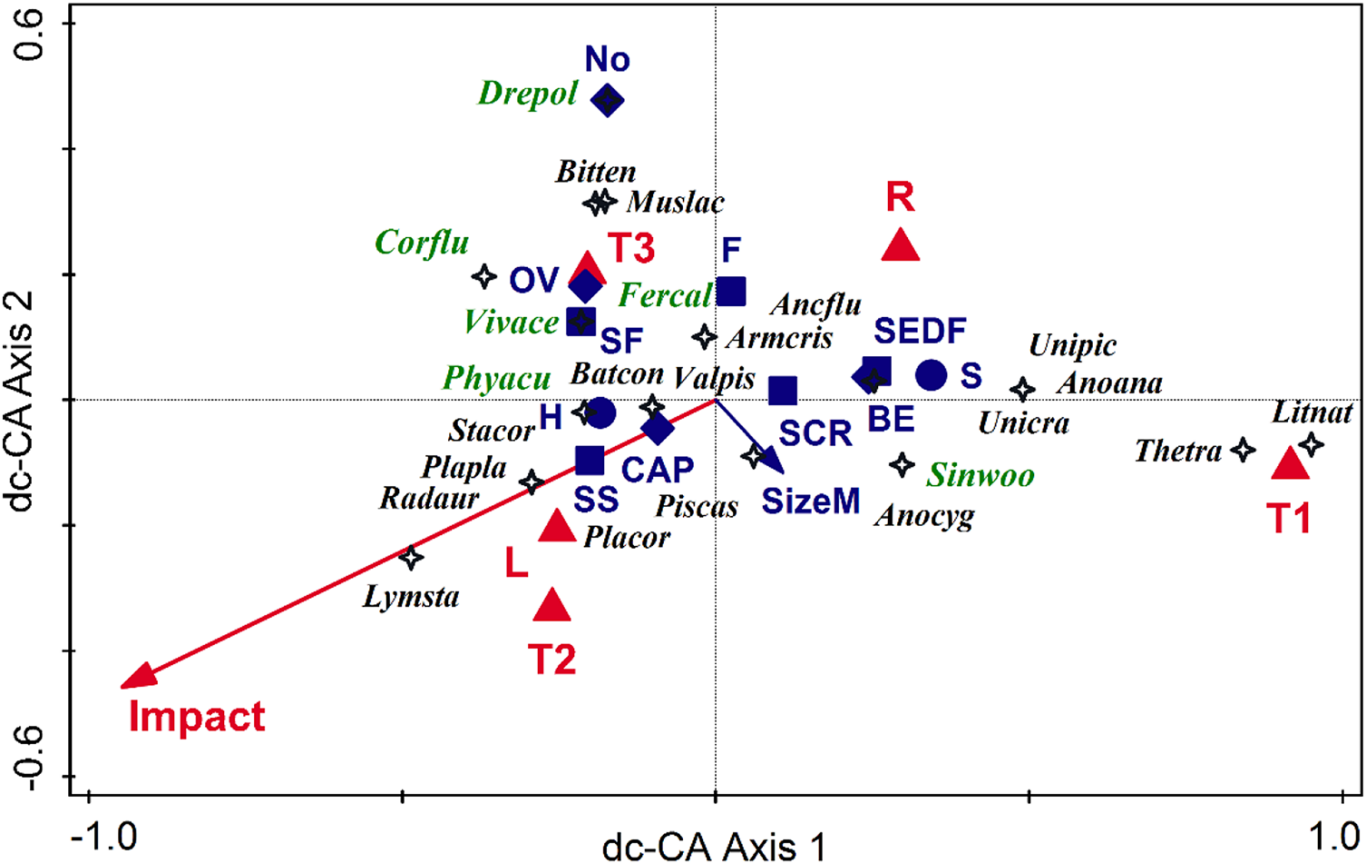
Double-constrained correspondence analysis (dc-CA) with selected predictors on presence-absence data in T1–T3. The selected functional traits (in blue) are Sexes (circles: H—hermaphrodite, S—separate sexes), Feeding type (squares: SCR—scraper, SS—scraper and sediment, SF—scraper and filter, F—filter, SEDF—suspension and deposit feeder), Oviposition (diamonds: OV—ovo-viviparity, CAP—capsule/eggmass, BE—parental care, juveniles in brood pouches of demibranchs, No—no oviposition, external fecundation), and mean body size (SizeM); the selected environmental predictors (in red) are time (Period, with levels T1—XIXth century, T2—1995–2000, T3—2020), habitat (R—river, lotic sector; L—lake, a lentic sector in reservoirs) and human impact (Impact). Species are coded by the first three letters of the genus and species names. The adjusted explained variation was 16.47%, the first axis accounts for 12.7% and the second for 2.2%. Native species have black labels, while aliens (AIS) are written in green.

In the dc-CA with the selected predictors on T1–T3 presence-absence data, the first two axes separate the communities by period, each positioned in a distinct quadrant (Fig. 2). After a decrease in diversity from T1 to T2, in T3, there were more species and higher functional diversity. In time, there was a reduction in body size, a switch from species with separate sexes to hermaphrodites, a transition of oviposition towards ovo-viviparity (in snails), and external fecundation (in bivalves), and a switch of the feeding type. The dc-CA adjusted explained variation was 16.47%; tests based on sectors and species showed significant relationships (combined test for all axes, pseudo-*F* = 2.6, *p* = 0.006), the dimensionality test based on case scores was significant for the first axis (pseudo-*F* = 4.2, *p* = 0.001) and marginally significant for the second one (pseudo-*F* = 1.1, *p* = 0.053). In contrast, the dimensionality test based on species scores was significant only for the first axis (pseudo-*F* = 1.6, *p* = 0.004). The adjusted variation explained by environmental predictors (Hab, Impact, and Period) was 28.36%, and by the selected functional traits (Sexes, FeedT, SizeM, and Ovipos) was 14.64%.

**Figure 3:**
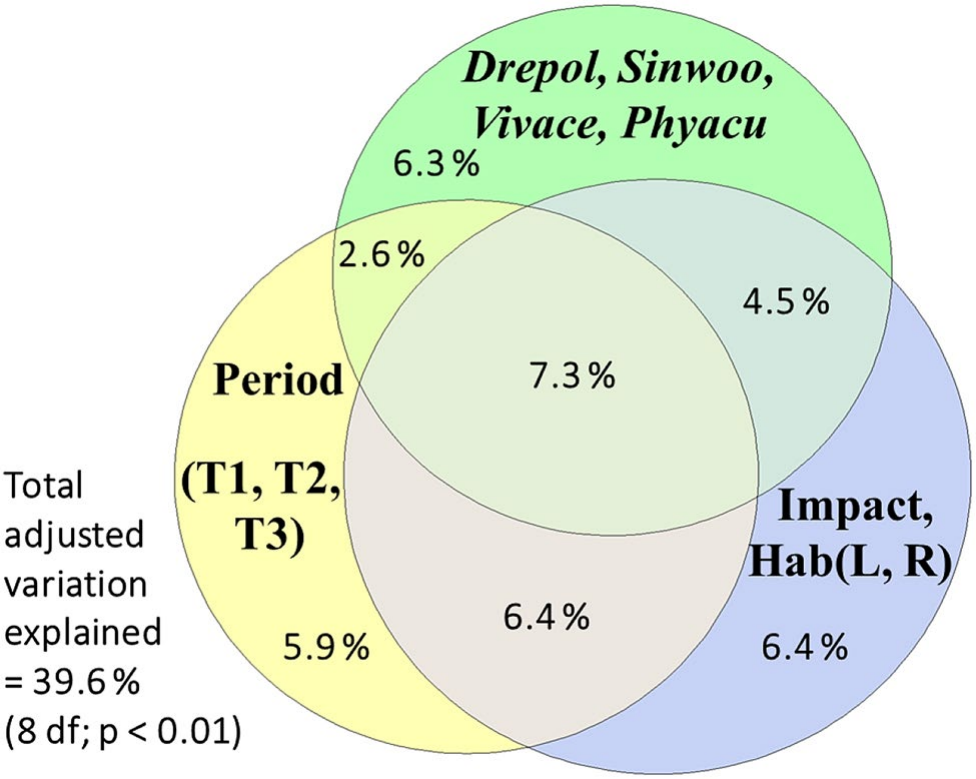
Structure of native communities explained by variation partitioning among time (Period: with levels T1—XIXth century, T2—1995–2000, T3—2020), environmental features (human impact—Impact, and habitat—Hab: R—river, lotic sector; L—lake, a lentic sector in reservoirs) and the selected alien invasive species—AIS (Drepol—*Dreissena polymorpha*, Sinwoo—*Sinanodonta woodiana*, Vivace—*Viviparus acerosus*, Phyacu—*Physa acuta*). RDA with hierarchical permutation scheme was used, interactive forward selection of predictors; both simple and conditional effects are significant (*p* < 0.01).

We have split the binary data describing communities into two parts: natives and AIS, using the latter as predictors. We partitioned the variation in native species composition explained by the three predictor groups (Period, Environment, and AIS) (Fig. 3), subjecting the explanatory variables to an interactive forward selection procedure. We used RDA with centered response variables (CCA can not be used because the empty rows in some tables hinder the use of a proper hierarchical permutation scheme). The adjusted explained variation was 39.6% (the simple effects: time accounted for 22.33%, habitat and impact 24.73%, and the selected AIS 20.82%). All simple and unique effects were significant (*p* < 0.01). Among the invasive species, *S. woodiana, V. acerosus,* and *P. acuta* had a significant effect, while *D. polymorpha* had a marginally significant effect (*p*_adj_ = 0.08). The unique effects were similar, with small differences between the environmental predictors (6.4%), AIS (6.3%), and time (5.9%). The highest value was registered for the shared effect of all predictors (7.3%), indicating a certain correlation between them.

### Actual (T3) mollusk communities' response to the environment, space, and alien invasive species

**Figure 4:**
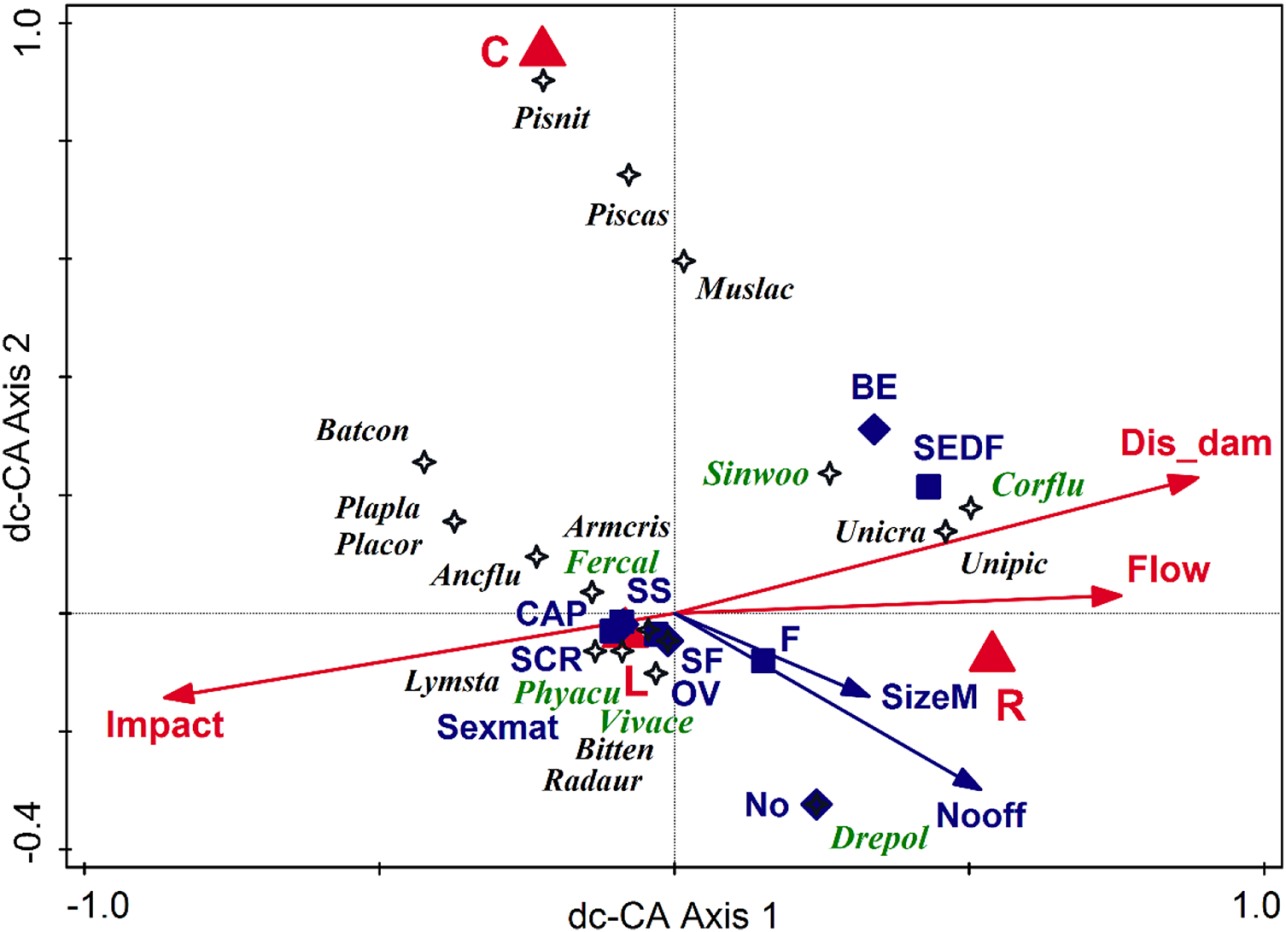
Double-constrained correspondence analysis (dc-CA) on species counts in 2020 (T3), with selected environmental predictors (in red: Dis_dam—distance to the nearest dam downstream; Flow—average water flowing conditions; Impact—human impact intensity; Habitat: C—canals, R—river, lotic sector, L—lake, lentic sector in reservoirs) and functional traits (in blue: SizeM—mean body size; Nooff—number of offspring; Sexmat—sexual maturity; Feeding type, as squares: SCR—scraper, SS—scraper and sediment, SF—scraper and filter, F—filter, SEDF—suspension and deposit feeder; Oviposition, as diamonds: OV—ovo-viviparity, CAP—capsule/eggmass, BE—parental care, juveniles in brood pouches of demibranchs, No—no oviposition, external fecundation). Species are coded by the first three letters of the genus and species names. Selected 18 species are depicted. The adjusted explained variation was 36.6%, the first axis accounts for 11.8% and the second for 10.4%. Native species have black labels, while the aliens (AIS) are written in green.

The following results refer only to species counts data from 2020. In the dc-CA on all species (Fig. 4) with selected environmental predictors (Dis_dam, Flow, Impact, Hab) and functional traits (SizeM, Nooff, Ovipos, FeedT, Sexmat), adjusted R^2^ = 42.64% for functional traits, adjusted R^2^ = 33.95% for the environmental predictors, and dc-CA adjusted R^2^ = 36.6% (combined test on sectors and species on all axes pseudo-*F* = 5.3, *p* = 0.001, on first axis pseudo-*F* = 0.5, *p* = 0.026). The effects of all selected predictors were significant (*p* < 0.05). The dimensionality tests based on case scores and on species scores were significant for the first four axes (*p* < 0.04). The human pressure rises in reservoirs toward the dams. It is associated with species having reduced number of offspring, reduced sizes, and oviposition in egg capsules, grouping the lentiphilic, pulmonates, and phytophilic species on the left side of the figure. The right side groups are mainly bivalves, rheophilic species, breeders, and species with an increased number of offspring and large body size. Some clams (Pisiidae) prefer canals, while naiads (Unionidae) occur in larger habitats, especially the riverbed.

We split the response data table into two components: natives and AIS. In the CCA with native species, the conditional effects of habitats Hab.C (canals) and Impact were significant (Table S1). For AIS, we used RDA since their gradient is short (2.8 standard deviation units), requiring a linear instead of a unimodal model. In the RDA with AIS, the conditional effects of Dis_dam and Flow were significant (according to the *p*-adj values, they were marginally significant, i.e. *p*-adj < 0.096). Thus, natives and AIS share no common predictors, indicating that the species composition of native and alien communities is linked to different environmental drivers (Table S1).

Analyzing relations between functional diversity and human pressure (Fig. S1), we have learned that AIS communities show a monotonic increase of functional diversity with impact, while the autochthonous communities respond by a sigmoid curve, with an accelerated increase at mid-values of impact, followed at higher values by an asymptotical reduction and then a decrease.

**Figure 5:**
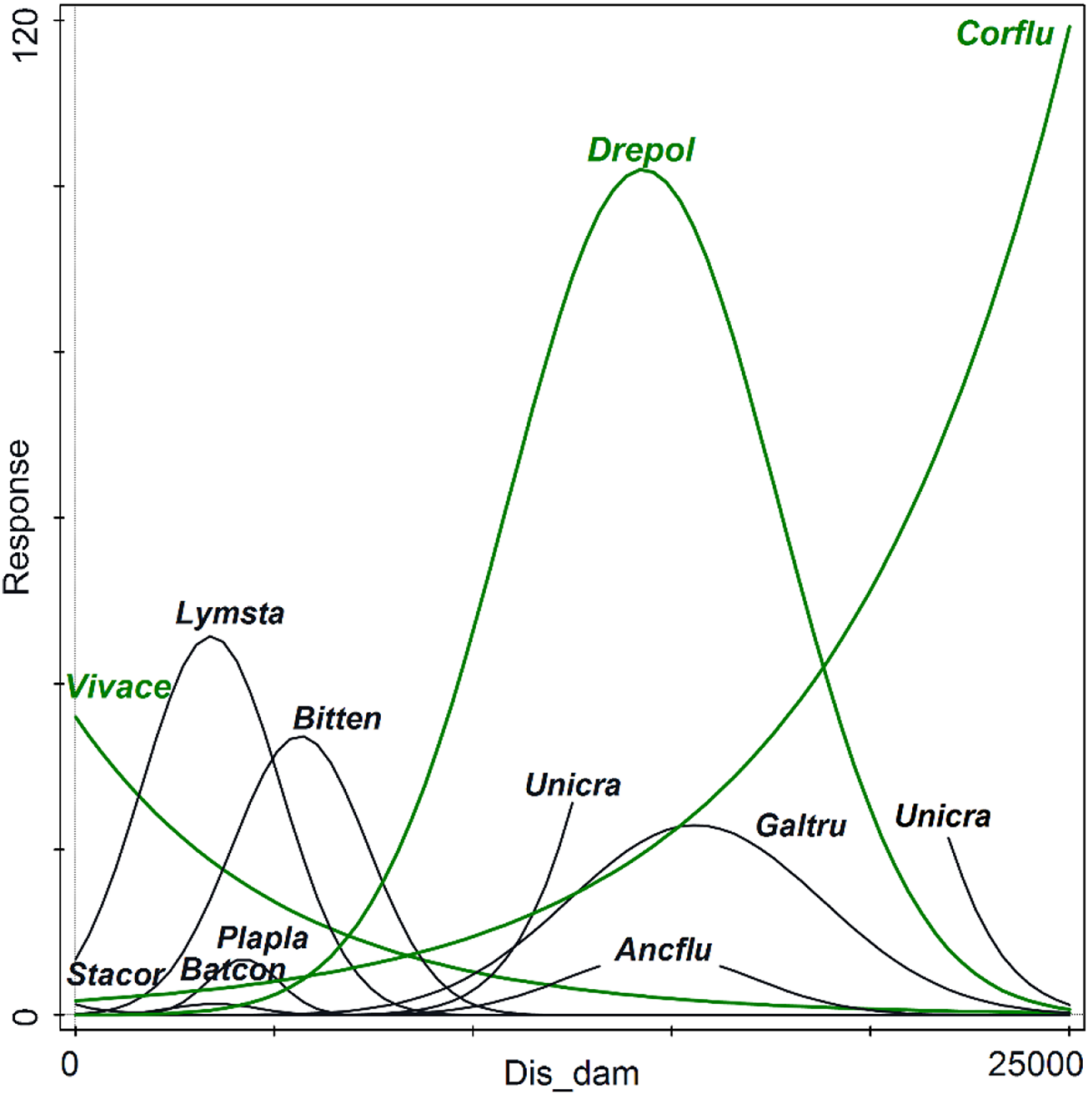
Species response curves to the Dis_dam: distance (in m) to dams (count data, generalized linear models, only a selection of significant models illustrated; the model parameters are given in Table S2; alien species are depicted in green).

Since dams (and reservoirs) are the most prevailing sources of human impact in the research area, and their effect is a major question the present paper addresses, we explored the species response curves related to the distance from the nearest dam placed downstream (Dis_dam), using GLM (Fig. 5, Table S2). Natives and AIS show mostly independent segregation of abundance curves in response to continuously modified conditions along this gradient (flow, sediment deposits, presence of plants along the banks), resulting in the coexistence of ecologically heterogeneous elements. There is a continuous range of responses, adaptations, and tolerances to the conditions associated with the flow and distance to the dam. There are no apparent groups of species but rather overlapping distributions of counts related to the gradient.

**Figure 6:**
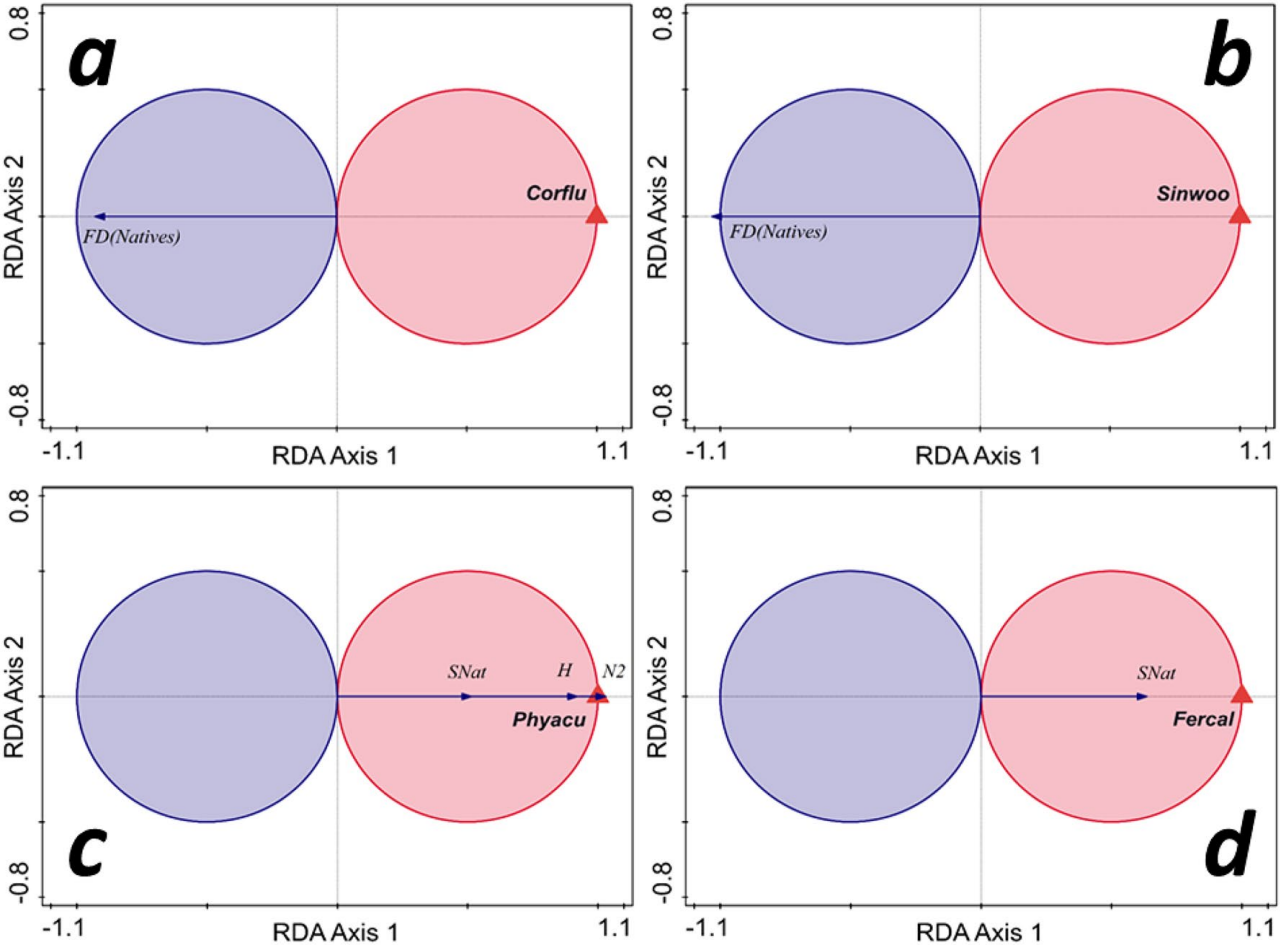
The t-value biplot (Van Dobben circles) for the response of Rao functional diversity of the native communities (FD(Natives)) to alien invasive species counts (AIS), with (**a**) significant negative effect of *Corbicula fluminea* (Corflu) and (**b**) marginally significant effect of *Sinanodonta woodiana* (Sinwoo), and for the response of taxonomic diversity measures (SNat—number of native species, H—Shannon index, N2—Hill's measure) of native communities in relation to AIS counts; two species of gastropods, (**c**) *Physa acuta* (Phyacu) and (**d**) *Ferrissia californica* (Fercal) had a significant positive effect. All the other relations of response variables with AIS were not significant.

We explored relationships between FD of the native community and AIS counts. FD(Natives) computed on all traits was found to be negatively related to the abundance of only two invasive bivalves. The relationship was significant for *C. fluminea* (pseudo-*F* = 4.6, *p* = 0.045, r^2^-adj = 16.0%), and marginally significant for *S. woodiana* (pseudo-*F* = 3.7, *p* = 0.062, r^2^-adj = 12.62%) (Fig. 6a,b). In contrast, taxonomic diversity measures of native community (Fig. 6c,d) were positively and significantly related only to two gastropods: *P. acuta* (SNat and H; pseudo-*F* = 12.8, *p* = 0.001, r^2^-adj = 38.23%) and *F. californica* (SNat; pseudo-*F* = 8.1, *p* = 0.006, r^2^-adj = 27.17%). All the other relations with AIS were not significant.

We also performed a variation partitioning of H and A, using E and S as explanatory variables, and a reverse analysis by inversing the roles of these matrices (Table S3). The null hypothesis states that the response variables are similarly distributed against the classes of variance explained by the predictors.

(1) A and H as response variables: the test statistic *χ*^2^ is 13.18 if A is the expected distribution and 16.17 if H is the expected distribution. Both are higher than the critical value for df = 3 and *p* = 0.01, thus, the two distributions are significantly different. Both A and H are better explained by the unique effect of S, followed by the unique effect of E, and then by the shared effect of S and E, but the difference is given by the residual variance, which is higher, almost double, in A (26.9%) than in H (14.1%).

(2) E and S as response variables: the *χ*^2^ test statistics (0.62 in both analyses) is less than the critical value for df = 3 and *p* = 0.05, thus, the two distributions are not significantly different. Natives and AIS predict the distribution of the variability in environment and space in the same manner: unexplained variation is highest in E and S, followed by the unique effect of H, the shared effect of H and A, the smallest values being estimated for the unique effect of A.

**Figure 7:**
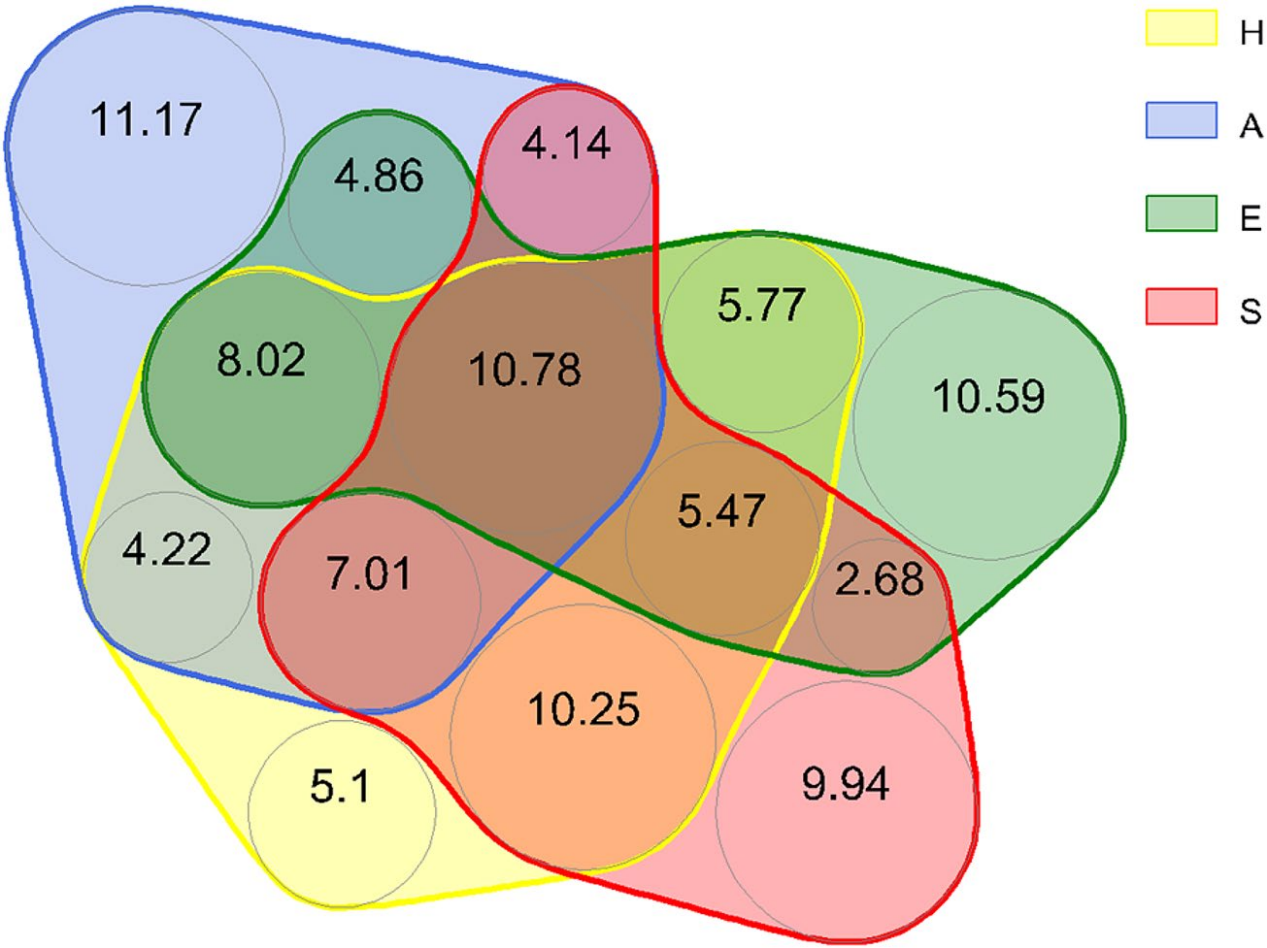
CUVARP diagram (Cumulative Variation Partitioning with Multiple Response and Predictor Matrices). All datasets are synthesized through selected PCoA axes scores of H—native species, A—alien invasives, E—environmental parameters, and S—space. No overlapping colored circles depict unexplained (residual) variation. The percentage of variation (explained and residual) from the total variation in all matrices is shown (the null value of AES overlap is not represented). Graphic alternatives are given in the Supplementary Information.

The complete extended novel algorithm for the cumulative variation partitioning (CUVARP) is given in the Supplementary Information. The algorithm for partitioning the variation in the native communities (H) explained by alien communities (A), environment (E), and space (S) is synthesized in Table S4, and intermediary results are given in Table S5 and illustrated in Fig. S2 to S5. The percentages (CUVARP diagram) are represented in Fig. 7 and Fig. S6.

The total variation in our data (PCA of all matrices—H, A, E, and S) was 160.0, of which the unexplained variation (residual variation of selected PCoA axes of H, A, E, and S) was 56.442 (35.28%). The explained variation was 103.558 (64.72%). The unexplained variation in the AIS community was higher than the corresponding part of the natives, indicating relative independence of AIS from the measured spatial and environmental predictors. The unique variations of A related to E and S were also lower than those corresponding to H.

A variant of this method also includes the FT (for the natives and the aliens), and it is introduced and depicted in Fig. S7 as the CWM-AVARP (Community Weighted Means—Average Variation Partitioning with Multiple Response and Predictor Matrices) diagram.

## Discussion

Old faunistic or ecological quantitative data are rarely embedded within the updated statistical analysis of ecological communities. This situation is primarily due to different approaches and differences in sampling design. By restoring historical information from XIX^th^ century publications and museum collections as simple presence-absence data, relating them to river sectors, and then relating these data to newly acquired information, we demonstrated that old ecological data, which is usually only briefly mentioned in the historical or introductive section of publications, can and should be transformed into dynamic and modern sources of functional ecological information, such as multivariate statistical models. The results reveal the dynamics of communities' responses to environmental changes and biotic drivers, such as invasive species and human pressure (Figs. 1a,b, 2).

Binary data may be used to assess shifts over time in community compositions^49^, when no quantitative data are available, when research questions are related explicitly to occurrences, or when sampling methodologies used in different studies are inconsistent. This practice might resurrect the importance and use of binary-based multivariate techniques. The recent development of new measures for quantifying directional changes in presence-absence community data^50^ results from this emerging need.

River damming is usually associated with negative impacts on communities' features, especially their diversity. However, we learned that the number of species has increased over time, reflecting the diversification of habitats (lotic, lentic, and intermediate) and communities, but also the colonization by AIS. These and the ongoing pollution reduction during the last decades^19,37^ support diverse and continuously dynamic communities adapted to various habitats and environmental factors. If the entire impacted area is considered, with all dams and lakes, flowing sectors, and canals, these provide a heterogeneous riverscape with a wide range of habitats. A few decades after the last dam was built, all conditions allow communities to reach advanced successional stages. The results show that the meta-community is enriched in species number and has a higher overall taxonomic and functional diversity, despite the extinction of some native species, interrupted longitudinal range of rheophilic taxa, and shifts in trait composition.

The T1–T3 binary sector-by-species data table has been related to a minimum of predictors since assessing environmental factors from two centuries ago is highly problematic. The time-related dc-CA (Fig. 2) shows that the human impact has increased from T1 (and from lotic sectors) to T2 (and towards the reservoir habitats). In contrast, it has decreased during the last two decades, placing the species in the ordination space in a triangular group, with their centroids aggregated towards T3. Lotic habitats, low human impact, and distant (past) times are related to species with separate sexes, breeders (parental care, juveniles in brood pouches of demibranchs), and a mixture of feeding behaviors (i.e., scrapers, suspension filters, and deposit feeders). The overall size of the mollusks decreases in time, from T1 to T3. Dam lakes and increased human impact (T2) are associated with hermaphrodites, oviposition in capsules (egg masses), and mixed scraper and sediment feeding. T3 is characterized by a reduced effect of impact, increasing species number, reduced body size, ovo-viviparity, external fecundation, and prevalence of filters and scrapers. While the mean number of native species per site increases almost linearly with time, the AIS increase by a polynomial model, supporting the "invasional meltdown" model^51^, which states that a cumulative number of AIS supports the invasion process, to which the facilitative interactions between the invaders are added. In the dc-CA on counts for present-day communities (Fig. 4), while many of the features formerly described on time-related binary data (Fig. 2) hold true (e.g., relations between species, traits, habitats, and impact), some novel selected variables and their associations lead to an increased resolution of ecological characteristics of newly established communities. Opposed to human impact is the distance to the downstream nearest dam and the river's flow. Both are positively correlated with the overall body size and the number of offspring. The AIS are distributed in the entire ordination space, each located close to a different group of native species. The six AIS are found mostly in reservoirs (lakes), on vegetation (*F. californica*) near the banks, near the shores or banks (*P. acuta*), or in the benthos (*V. acerosus*). They can live both in lotic sectors and dam-lakes, attached to hard substratum (*D. polymorpha*), in sediments (*S. woodiana*), or less abundant in lakes but mainly in lotic, sandy sectors (*C. fluminea*). Thus, the AIS use differentially the available habitats and resources, covering the entire range of ecological conditions and traits space, being placed closer to distinct groups of natives but distant to each other in the ordination space. This insight might lead to an invasive niche differentiation and an adaptive strategy to reduce the intra-group (AIS) competition for resources.

Analyzing responses of native and alien fish species in dam reservoirs, Muniz et al.^10^ used variation partitioning to estimate the effects of different predictor matrices on one or the other community component, which show mostly similar, but also some different responses to the predictors. Studying drivers of freshwater fish richness across North America, Anas & Mandrak^52^ showed that environmental and anthropogenic factors were more important than historical factors in explaining native and alien richness. However, different environmental drivers influence the two richness patterns. These findings are partially consistent with ours, but we also showed differences in relationships of the native versus the AIS community components (Table S1) that we interpret as a possible explanation of invasive success. Our results show that the species compositions of native and AIS communities are explained by different environmental predictors, while the unexplained variation in the community structure is larger in AIS compared to natives (Table S3).

The native assemblages' functional diversity varied negatively and significantly with the counts of the newly colonized *C. fluminea*—probably a colonist in the river since a few years before its first finding in 2020— and marginally significantly with *S. woodiana* (Fig. 6a,b), which colonized the river more than a decade ago. This finding might hint at an increased negative impact of *C. fluminea* compared to other AIS. In contrast, some taxonomic diversity measures were significantly and positively predicted by only two gastropod AIS (Fig. 6c,d), *P. acuta* and, to a lesser extent, *F. californica*. We stress that this result should not be interpreted as AIS necessarily causing these variations in diversity. It shows that sites where those AIS are present and more abundant, differ in respect of native communities' diversity. In our study, the positive relations of taxonomic diversity with some invasive gastropods is probably the result of river damming, which lead to the diversification of bank and periphytic assemblages and life conditions, favoring gastropods, and also the increase of diversity of lentic ecosystems. However, damming also resulted in the debasement and functional reduction of lotic ecosystems and habitats, hence the negative relations of native communities' functional diversity with the two alien bivalves. The combined effect of river degradation and the introduction of AIS are drivers for native species loss and changes in the traits space^49^, leading to a dominance of invaders and homogenization of communities' structure.

The multivariate approach is essential for understanding freshwater mollusks' distribution and other ecological parameters^53^. However, we showed that understanding the changes and responses of native freshwater mollusk communities requires more than a multivariate approach. All dimensions (i.e., matrices describing and characterizing a source of explanatory variability), including time, space, environmental features, traits, and distributions of AIS (and undoubtedly many more), have their significant predictive role in explaining how native communities adapt, respond, and change. Therefore, a multidimensional approach is needed, and here we propose a simple algorithm for such an analysis. The newly developed CUVARP analysis and diagram (Fig. 7) indicate a different relationship of natives and AIS with environmental and spatial predictors and suggest a possible explanation for the success of invasive over the native communities.

Both the CUVARP diagram and results from Table S3 show that the unexplained (residual) variability in AIS is significantly larger (about double) than in native communities. Besides, the relative dependence of natives on environment and space is higher, while AIS are less related to the measured predictors (lower explained variation). This may be due to broader ecological niches and tolerance of AIS to environmental gradients, conferring advantages in colonizing, exploiting, and competition strategies. It also hints at their invasion success and ability to outcompete natives. Conversely, residual (unrelated) variance in space and environment might be used in further studies to investigate how other communities exploit and compete for the available resources and conditions, which might bring insight into metacommunity structural and functional features. Variability of conditions, range of fluctuations, and abundance of resources are also limited; thus, asking the reverse question (how life explains external variables and data tables) might be legitimate. Such analyses might show and test different relations at higher levels (guilds, metacommunity, supracommunity)—how communities are organized and structured, how they interact and function, and how they (co)adapt to external conditions and exploit limited resources. Therefore, we also advance the idea that the so-called "residual variation" is a possible valuable source of information and requires further consideration concerning its meaning in community ecology.

Another idea in our framework is the necessity and possibility of analyzing variation partitioning both among predictors (as usually done) and at the level of coexisting and interacting communities (or response matrices). Understanding interactions between communities and their relations to external descriptors also requires analysis of their simple and conditional responses, meaning the discrimination between their unique and shared explained variance related to the same predictors. Thus, our approach introduces the necessity of finding ways of a complete variation partitioning, meaning measuring, testing, and illustrating both unique and shared effects, in matrices (data tables, arrays) of predictors as well as responses, including all related data sets, disregard of their nature, domain, type of data and role. Our algorithm may be a small and feeble step in this frame, but it shows the urge to move forward.

A shortcoming of the CUVARP analysis, is that this framework might explore and test relations and effects of groups of predictors but cannot explore the effects of interactions within the classes. For example, understanding interaction between invaders is crucial for the outcome of their effect at the community level, needing a detailed consideration of their neutral, positive, and negative interspecific feedback during invasions^54,55^. On the other hand, monitoring trait dynamics within a multi-matrices frame might reveal changes in life history, e.g., evolution induced by interspecific relations between native and AIS, as sometimes revealed at a short timescale^56^. A step forward is embedding functional traits in our algorithm, using CWM instead of sites-by-species data tables, and producing cumulative (CWM-CUVARP) or average diagrams (CWM-AVARP), the last shown in Fig. S7.

Lopes-Lima et al.^28^ reviewed the current knowledge on diversity and conservation of freshwater mollusks defining and using eight knowledge shortfalls, which we also endorse for other taxonomic and ecological groups (related to the taxonomy, distribution, population parameters, evolution, ecological valences, traits, biological relations, and conservation). We expand this framework by identifying two more knowledge gaps: (9) the ecological knowledge gap (ranging from communities to ecosystems), which we term the Odum Shortfall (after Eugene P. Odum), and a synthetic approach, namely (10) the mathematical-informatical system of linking multiple data sets from many dimensions, for analysis, illustrative methods and interpretative tools, which we call the Hawking Shortfall (in honor of Stephen Hawking). Our work adds a new step by addressing the problem of multiple response matrices (in our case, native and AIS components of freshwater mollusk communities) linked to multiple predictor data tables. The applications are numerous: the most pragmatic are linked to the conservation of native species and communities, management of invasive species, land use, and riverscape research and management.

To understand the big picture, we conclude that a transition in methodology is required for linking and comparatively analyzing native and AIS communities while simultaneously considering their relations to the environment, space, human pressure, functional traits, and other data sources. This means using an unlimited number and types of explanatory data tables constraining sites and species (rows and columns). Possible alternatives to these techniques consist of newly developed methods for quantifying and comparing n-dimensional hypervolumes^57^. Here we demonstrate a methodology that can accomplish these goals by transitioning from "variables to matrices" and from "multivariate to the multi-matrix statistical modeling", some elements that support what we have earlier termed as an "omni-spaces explanatory communities ecology"^45^.

## Supplementary Information

Supplementary Information.

## Data Availability

The datasets generated and analyzed during the current study are available in the KNB repository: "Ioan Sîrbu, Ana Maria Benedek, and Monica Sîrbu. 2022. Data on the freshwater mollusk communities, environmental parameters, functional traits, niche, and spatial coordinates, from the middle Olt River (Romania). Knowledge Network for Biocomplexity. 10.5063/F1TQ5ZZ5".
