# Supplementary material for: Disentangling structural and functional responses of native versus alien communities by canonical ordination analyses and variation partitioning with multiple matrices
Source: Sci Rep. 2022 Jul 27;12:12813. doi: 10.1038/s41598-022-16860-6 (PMC9329446; doi:10.1038/s41598-022-16860-6)
Supplement: Supplementary file 1 — Supplementary Information. [file 41598_2022_16860_MOESM1_ESM.pdf]

Scientific Reports

Disentangling structural and functional responses of native versus alien communities by canonical ordination analyses and variation partitioning with multiple matrices

Ioan Sîrbu, Ana-Maria Benedek, Bryan L. Brown, Monica Sîrbu

Corresponding author: Ioan Sîrbu, Lucian Blaga University of Sibiu, Faculty of Sciences, 5-7 Raţiu Street, 550012 Sibiu, Romania, ioan.n.sirbu@ulbsibiu.ro, meosirbu@yahoo.com

Supplementary Information

Table S1. Simple and conditional term effects of environmental predictors on counts of native and alien invasive (AIS) freshwater mollusk communities. Degrees of significance are according to the *p*-value and the value adjusted by false discovery rate—p(adj). In the native community there was high species turnover (the length of gradient was over 4 standard deviation units), therefore we used the unimodal canonical correspondence analysis (CCA), while the AIS had a low species turnover, thus we used the linear redundancy analysis (RDA).

CCA of native species counts explained by environmental predictors									
Simple Term Effects:					Conditional Term Effects:				
Name	Explains %	pseudo-F	p	p(adj)	Name	Explains %	pseudo-F	p	p(adj)
Hab.C	17.2	3.7	0.011	0.014	Hab.C	17.2	3.7	0.019	0.048
Impact	16.1	3.5	0.001	0.003	Impact	15.5	3.9	0.001	0.005
Hab.L	15	3.2	0.008	0.013	Hab.R	6.2	1.6	0.13	0.217
Flow	13.9	2.9	0.001	0.003	Hab.L	6.2	1.6	-	-
Hab.R	12	2.4	0.038	0.038	Flow	2.8	0.7	0.589	0.704
Dis_dam	9.6	1.9	0.089	0.089	Dis_dam	2.6	0.7	0.704	0.704
RDA of AIS counts explained by environmental predictors									
Simple Term Effects:					Conditional Term Effects:				
Name	Explains %	pseudo-F	p	p(adj)	Name	Explains %	pseudo-F	p	p(adj)
Dis_dam	18	3.9	0.029	0.048	Dis_dam	18	3.9	0.019	0.095
Impact	13.7	2.9	0.02	0.048	Flow	11.6	2.8	0.038	0.095
Flow	13.2	2.7	0.025	0.048	Impact	5.6	1.4	0.271	0.452
Hab.R	12.2	2.5	0.041	0.051	Hab.C	3.1	0.7	0.542	0.678
Hab.L	10.5	2.1	0.055	0.055	Hab.L	0.8	0.2	0.957	0.957
Hab.C	2	0.4	0.736	0.736					

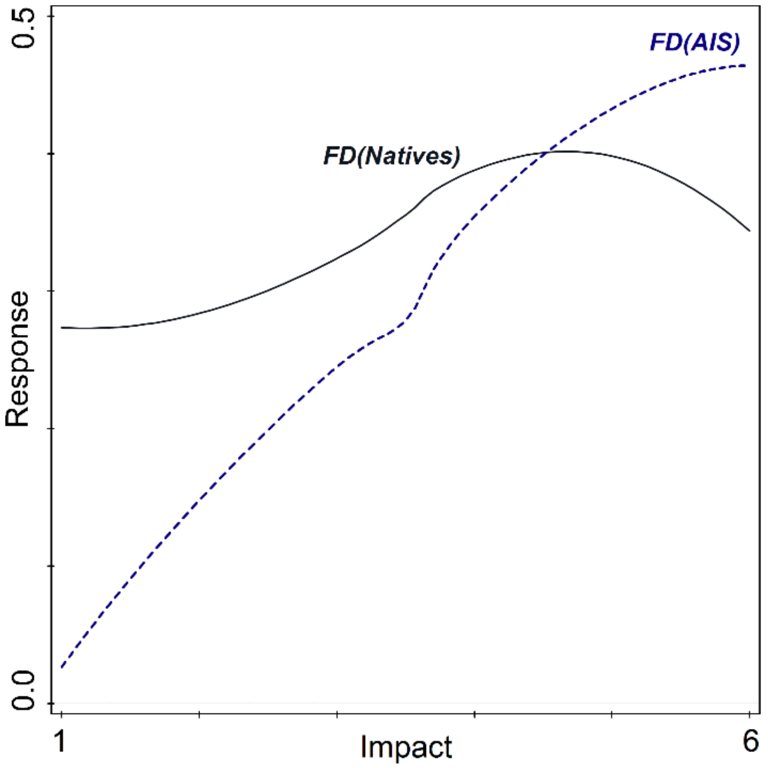

Fig. S1. Functional diversities of natives—FD(Natives)—and of alien invasive species—FD(AIS)—related to Human Impact (response curves, loess models)

Table S2. Parameters of species response to the distance to nearest downstream dam (Dis_dam), from Fig. 5, described by cubic (or less) polynomial generalized linear models (GLM), with Poisson distribution, accounting for overdispersion; stepwise selection was performed based on the F parameter, at α = 0.05.

Summary of fitted Generalized Linear Models:				
Predictors	Dis_dam			
Distribution	quasi-Poisson			
Link function	log			
GLM fitted for 15 response variables:				
Response	Type	R^2(%)	F	p
Vivace	linear	27.3	6.1	0.023
Bitten	quadratic	37.7	4.3	0.033
Galtru	quadratic	41.2	3.9	0.040
Stacor	linear	69.3	49.6	< 0.001
Lymsta	quadratic	39.4	3.7	0.051
Placor	linear	24.6	5.1	0.035
Plapla	quadratic	56.4	11.1	0.001
Batcon	quadratic	55.4	6.5	0.009
Armcris	quadratic	53	4.4	0.050
Ancflu	quadratic	55.4	6.9	0.006
Unipic	cubic	100	3402	< 0.001
Unicra	quadratic	54.5	8.6	0.002
Corflu	linear	48.8	6.7	0.018
Piscas	quadratic	77	9.3	0.031
Drepol	quadratic	33.1	3.7	0.046

Table S3. Variation partitioning of response variables and predictor data tables, using explanatory (EV) and response variables (RV) in turn: H and A (scores of selected native and alien invasive species Principal Coordinates Analysis—PCoA—axes); E and S (scores of selected environment and space PCoA axes). Upper values represent explained variation in RV, while the lower values indicate the percent of explained variation (unique and shared effects).

		**EV**				
		**Ø**	**E**	**E ∩ S**	**S**	**TOTAL**
**RV**	**H**	11.280<br>*(14.10%)*	9.787<br>*(12.23%)*	7.251<br>*(9.07%)*	11.682<br>*(14.60%)*	40<br>*(50%)*
	**A**	21.515<br>*(26.89%)*	6.107<br>*(7.63%)*	4.789<br>*(5.99%)*	7.589<br>*(9.49%)*	40<br>*(50%)*
		**EV**				
		Ø	H	**H ∩ A**	**A**	**TOTAL**
**RV**	**E**	19.617<br>*(24.52%)*	9.490<br>*(11.86%)*	7.547<br>*(9.44%)*	3.346<br>*(4.18%)*	40<br>*(50%)*
	**S**	17.514<br>*(21.89%)*	10.109<br>*(12.64%)*	8.825<br>*(11.03%)*	3.552<br>*(4.44%)*	40<br>*(50%)*

Detailed synopsis of the methods used for the analysis of the mollusk communities' time dynamics

Variables used. Historical data on species distribution in the middle Olt River have been restored by the revision of literature and collections from the XIXth century (T1). We have read and translated old literature written in German and found toponyms and localities using old military maps and dictionaries of localities since that territory had been part of different countries and administrative regimes. We ascribed the sampling points to 11 sectors of the 83 km reach of reference. Only data from the Olt riverbed were considered in this article, while those from wetlands and other water bodies were discarded. Presence-absence data from T2 (1995–2000) and T3 (2020) are based on our surveys and are, in this section, ascribed to the same river sectors. Restoring past predictors (explanatory variables) is difficult, so we focused on the minimum number and types. Period (factor with levels T1, T2, and T3) was one of them since the data are related to time. We set human impact (the second predictor) in T1 as the baseline (Impact = 0 for all sectors, meaning the natural status of the river), and we assessed the distance to the natural status for each sector in T2 and T3. The last predictor was habitat, with two levels: R for river (the single level for T1 since dams were built in the second half of the XXth Century) and L for lakes, the last being used for sectors defined by reservoirs.

Testing for significance. Data were arranged first by sector and then by time (Period), meaning that each sector (from 1 to 11) of the middle Olt River had three rows, one for each period (T1, T2, and T3). We used the Monte Carlo test with 999 permutations with a hierarchical design permutation scheme, considering the river's sector a whole-plot; thus, the number of split plots in each of the 11 whole-plots was 3 (corresponding to the periods). We selected the option of time series or linear transect for both whole-plot and the split-plot permutations. The predictors' effect on all constrained axes test was provided using pseudo-F value and probability. The adjusted explained variation is given in percents [1S, 2S].

Fig. 1a. Canonical correspondence analysis (CCA) on sectors-by-species binary variables data set with all 28 species, explained by Period, and tested as described above. When response variables are binary data, CCA is the recommended method.

Fig. 1b. CCA on sectors-by-species binary data (all 28 species), with Period, Impact (numeric), and Habitats (R or L) as predictors. We have chosen not to display the results as a CCA diagram with species but to project the functional diversity—FD(Rao)—isolines using a generalized additive model (GAM). The other features related to testing, hierarchical permutation scheme, and test results are identical to those explained before. The FD(Rao) diversity is based on two tables: the

functional traits (FT, species-by-traits table) and sectors-by-species data set. The original formula of Rao [3S] is used as explained in references [1S, 2S, 4S], this being a generalized form of the Simpson index of diversity. Functional diversity or trait variability can be directly computed on numerical variables (traits and species data table). The problem is that traits are usually a combination of quantitative, ordinal, and factorial, as in our study. The problem is solved by working it around (for details, see [1S] p. 159–160, 290–292; [2S] p. 463). First, Gower distance ([2S] p. 143, [5S] p. 278–280) was used for calculating a distance matrix between species on all FT, then a Principal Coordinates Analysis (PCoA) was applied to the matrix of distances, and the PCoA scores were saved as an independent matrix (data-table). Axes in a PCoA can be selected differently; we have opted for those with non-zero eigenvalues (one other alternative would have been the positive eigenvalues). The latter data set with scores was used in combination with the sectors-by-species matrix for computing the FD(Rao) values, these being plotted on the CCA ordination space diagram using a GAM, as explained in the main text (details and examples in [1S, 2S]). Details for the GAM: we used a Gaussian distribution, with stepwise selection using the Akaike information criterion (AIC) for model selection.

Fig. 2. Four tables were considered in the double-constrained correspondence analysis (dc-CA): the sectors-by-species qualitative data set (binary data on all species), the same data but transposed (as required by the method in Canoco software), the FT, and the Environmental predictors [2S, 6S]. We have used a procedure of forward selection of predictors. To avoid circular reasoning, we excluded some traits (such as Origin, Taxon, and Habitat). We made a bottom-up selection of predictors according to the p-values adjusted by the false discovery rate (FDR). The rest of the methodological details are given in the main text.

Fig. 3. Variation partitioning analyses. We have split the sectors-by-species binary data table in two, for natives and alien invasive species (AIS), the last being treated as one of the groups of predictor variables. The response data table was the native community (presence-absence values of native species only). We have explored and tested the unique and shared effects of three groups of predictors on the native communities, namely time (Period), environment (Habitat, Impact), and the AIS, while also applying an interactive selection of the predictors from each group. Testing was done as described above, using a 999 permutations Monte Carlo test with the hierarchical permutation scheme. Because the AIS have articles with zero values, CCA could not be applied because, in unimodal analyses, empty (null) rows are eliminated [1S], leading to an unbalanced design, which does not support a hierarchical permutation scheme. Because of this limitation, we applied its linear counterpart, a variation partitioning analysis based on the redundancy analysis (RDA) with a hierarchical design permutation scheme.

Detailed synopsis of the methods used for the analysis of actual mollusk communities' response to the environment, space, and alien invasive species

In this section, we have used only the actual (T3) quantitative (counts) data.

Testing for significance. We used the Monte Carlo test with 999 unrestricted permutations in all the following multivariate analyses.

Variables used. Response variables were sites-by-species counts from the year of 2020 surveys (T3 only). We have selected 20 sampling sites along the river's course, and their GPS coordinates in decimal degrees are given. In Fig. 4 and 5 we have used data on all species (the entire community) distinguishing between counts of the natives and AIS. The traits (FT) are the same as in the former section. Using the distance-based Moran Eigenvector Maps (db-MEM), the space coordinates (latitude and longitude) have been turned into axes scores [1S, 2S, 7S]. To the environmental predictors, we added Flow (ordinal variable assessing the velocity and type of water flow) and Dis_dam, the distance to the nearest downstream dam across the Olt River. In further analysis we have split the community data in two, counts of native and AIS, testing comparatively their response to environment and space.

Fig. 4. The dc-CA on four data tables: the site-by-species counts, its transposed form, the environmental variables and the functional traits, predictors selected using a bottom-up interactive selection procedure, based on p-values adjusted by FDR as criteria for significance. The aim was to investigate and test relations of actual species counts with external drivers and traits.

Table S1. Exploring relations between counts of natives and separately of AIS and the environmental factors while testing for the predictors' effects (both simple and conditional). Since the gradient was high in natives (5.3 SD units) and low in AIS (2.8 SD units), we used a CCA for the former and an RDA for the latter. Comparing the conditional terms effect, different predictors have been found significant for the two response data tables, meaning that natives and AIS respond to different environmental factors.

Fig. S1. Testing relations of functional diversities of natives and AIS with human impact. We have assessed the FD separately on natives and AIS on actual count data, similarly to the calculation of FD on time-related binary data. The values of FD for natives, FD(Nat), and alien invasives, FD(AIS), have been related as response variables to Flow, Dis_dam, and Impact as predictors in an RDA. The RDA was selected because the two functional diversities are not compositional; thus unimodal models (CCA) are not suitable. Response curves related FD to the Impact by loess functions. Model parameters were: local quadratic model, with a span of 1.0, and robust fitting algorithm. The determination coefficients for the two models were r^2 = 26.8% (standard error 1.118) for the natives and r^2 = 29.3% (standard error 0.241) for the AIS.

Fig. 5 and Table S2. As an attribute plot of the CCA of all species counts and environmental factors as predictors, we have revealed the relations between the species response to an important driver in our study, namely the distance to the nearest downstream placed dam (Dis_dam) expressed in m. We have used generalized linear models (GLM); the parameters, significance results, and other statistics are given in Table S2. The species that revealed non-significant relations to Dis_dam were discarded. The species response curves were constructed by selecting Dis_dam as a predictor and all species, in turn, as response variables with Poisson distribution. The linear, second, and third-order polynomial models were tested (results are given in Table S2) by F-test statistics with an alpha level of 0.05, using the quasi-distribution approach to account for overdispersion.

Fig. 6. We have tested relations between diversity measures (both functional and taxonomic) of natives and the counts of AIS (as predictors) by RDA. The significant results are shown as t-value biplots (also called Van Dobben circles), showing the response variables related to the AIS counts. When the arrow (quantitative response variable) ends within the red circle, the response is positive and significant. When it ends in the blue circle, the response to the particular predictor is negative and significant. At the edge, a marginally significant result might be inferred. Figure 6 depicts the response of FD(Natives) to AIS, with (a) significant negative effect of *Corbicula fluminea* (Corflu) and (b) marginally significant effect of *Sinanodonta woodiana* (Sinwoo). We tested the response of taxonomic diversity measures (SNat—number of native species, H—Shannon index, N2—Hill's measure) of native communities to AIS. Two species of gastropods, (c) *Physa acuta* (Phyacu) and (d) *Ferrissia californica* (Fercal), had a significant positive effect. All the other relations were not significant.

Table S3. This analysis is part of the novel methodology introduced with this article, in which PCoA turns original data tables into axes scores using Gower or Euclidean distances as presented before. The scores were further used, in turn, as response and predictors, and a selection of significantly related axes from each table was made. The entire algorithm is given below within the CUVARP section. In this table, variation partitioning of predictor data tables is accomplished, using explanatory (EV) and response variables (RV) in turn: H and A (scores of selected native and AIS PCoA axes); E and S (scores of selected environment and space PCoA axes). Principal Components Analysis (PCA) assessed the total variation in each matrix on centered and standardized variables. Since the response variables are not compositional, relations between response and predictor matrices were analyzed using RDA on centered and standardized variables. The aim was to test if native and AIS communities experience differences in their relations to space and environmental predictors.

Performing an inverse analysis, with species as predictors, we explored their ability to predict variation in the space and environment, as responses.

Fig. 7 and the CUVARP algorithm and diagrams are explained below.

Algorithm for cumulative variation partitioning with multiple response and explanatory matrices (CUVARP) analyses and diagrams

We aimed to introduce a new method for a complete and cumulative variation partitioning, in which the data tables were repeatedly switched so that each became a response data set. The simple and conditional effects of the others were explored in turn, resulting in a complete partitioning of all effects. These were summed and expressed as percentages from the total variation in all data tables. The residual variation in each matrix was also assessed, and the resulting values are represented in the newly introduced diagram.

Step 1. Summarizing the data; the algorithm uses the following tables:
(1) sites-by-native species (we have used counts, but occurrence or dominance can also be used);
(2) sites-by-AIS species (counts of AIS were used in our study);
(3) sites-by-environmental variables;
(4) sites-by-spatial coordinates (i.e., latitude and longitude, but in other studies, elevation, depth, etc., might also be considered).

The first three tables are each subject to a PCoA on standardized and centered data using the Gower distance (in other studies, any other suitable distance might be used instead), and all axes with positive eigenvalues are used. The PCoA axes scores (site-by-scores) are saved as data tables named "H-all" (natives), "A-all" (alien invasives), "E-all" (environment). The last table (sites-by-spatial coordinates) is subject to a distance-based Moran Eigenvector Maps analysis (db-MEM), formerly named principal coordinates of neighbor matrices analysis (PCNM) [1S]. The cut-off threshold is determined by the nearest neighbor using the euclidean distance, and all axes with positive eigenvalues are used. The matrix of site-by-PCoA spatial scores is denoted "S-all."

Step 2. Selecting the variables (the PCoA scores from the four tables significantly linked to each other). Using, in turn, H-all and A-all as response data tables, with the other three tables of PCoA scores used as matrices of explanatory variables (within a variation partitioning analysis with three groups of predictors and interactive forward selection procedure), we did RDAs on centered response variables with predictor selection. By reaching a consensus between these analyses, we selected the axes scores significantly related to each other, these being termed H (natives, scores of PCoA axes 1 and 2), A (alien invasives, scores of PCoA axes 1 and 5), E (environment, scores of PCoA axes 1 and 6), and S (scores of PCoA axes 1 and 3).

Step 3. A PCA was done for the original matrices on standardized and centered variables to assess the total variability in each data table. We calculated the total variation in the data by adding these values. In our study, the total variation was 160.

Step 4. Each of the four data tables with selected axes scores (H, A, E, and S) was used in turn as a response matrix, while the other three served as explanatory data tables in variance partitioning analyses, accounting for a complete disentangling of simple and conditional effects, and also the evaluation of residual variation. The variation partitioning was done using RDA, with and without covariates, the response data being standardized and centered. We illustrate how this was done using H as response and A, E, and S as explanatory tables (Table S4). The Venn diagram of variation partitioning is given in Fig. S2. The analyses for a complete variation partitioning are given by a series of equations, which use: RDA followed by the name of the response data table (in this case H) and the tilde ~ followed by the tables with the selected predictors (e.g., A+E+S, where the sum symbol + is used if more then one predictor data table is used). Predictors from a table used as covariates are denoted by placing the table abbreviation after the vertical line |.

Besides assessing the explained variance, the predictors' simple, conditional (unique), and shared effects were also tested against the null hypothesis that their effect is random. We used the Monte Carlo permutation test with 999 unrestricted permutations. Simple effects can be measured

and tested using RDA without covariates, while the conditional effects can be measured and tested using the analyses with covariates. Not all shared variation parts can be tested for their significance. The formulas for calculating the variation parts involve the sum of canonical eigenvalues (coded as ceg) of the constrained analysis of predictor tables.

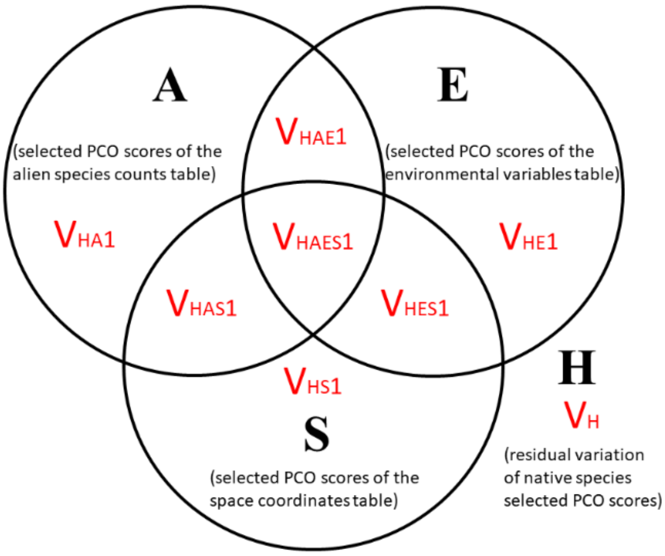

Fig. S2. Variation partitioning conceptual (Venn) diagram, showing the unique and shared effects of the predictors on the native community (selected PCoA scores are used for each matrix, H—natives, A—alien invasives, E—environment, and S—spatial eigenvectors), the number in codes corresponds to the current analysis. The corresponding values are given in Table S4.

Table S4. Algorithm for partitioning the variation in the native communities (H) explained by alien invasive communities (A), environment (E), and space (S). The tilde ~ stands for canonical ordination analysis, followed by the predictors. The total variation (Var(H)) is given by the PCA of H (PCA H). Canonical eigenvalues are coded as ceg, and the predictors are enclosed in brackets []. Vertical bar | separates the covariates (to its right), and the lowercase letters correspond to the variation parts given in Fig. S2. Significance tests on all axes are given.

Step	Analysis	Estimates	Significance tests (*p*) on all axes
1.1	PCA H	Total variation = Var(H) = 40.0	-
1.2	RDA H ~ A | (E + S)	V_{HA1} = Var(H) * ceg[A | (E + S)] = = 3.121	conditional effect of A, pseudo-*F* = 2.5, *p* = 0.073
1.3	RDA H ~ E | (A + S)	V_{HE1} = Var(H) * ceg[E | (A + S)] = = 3.276	conditional effect of E, pseudo-*F* = 2.6, *p* = 0.062
1.4	RDA H ~ S | (A + E)	V_{HS1} = Var(H) * ceg[S | (A + E)] = = 9.596	conditional effect of S, pseudo-*F* = 7.6, *p* = 0.004
1.5	RDA H ~ (A + E) | S	V_{HAE1} = {Var(H) * ceg[(A + E) | S]} - - V_{HA1} - V_{HE1} = 6.511	conditional effects of (A + E), pseudo-*F* = 5.1, *p* = 0.003
1.6	RDA H ~ (A + S) | E	V_{HAS1} = {Var(H) * ceg[(A + S) | E]} - - V_{HA1} - V_{HS1} = 2.086	conditional effects of (A + S), pseudo-*F* = 5.1, *p* = 0.003
1.7	RDA H ~ (E + S) | A	V_{HES1} = {Var(H) * ceg[(E + S) | A]} - - V_{HE1} - V_{HS1} = 1.902	conditional effects of (E + S), pseudo-*F* = 5.9, *p* = 0.002
1.8	RDA H ~ (A + E + S)	V_{HAES1} = {Var(H) * ceg(A + E + S)}- - V_{HA1} - V_{HE1} - V_{HS1} - V_{HAE1} - V_{HAS1} - - V_{HES1} = 5.350<br>V_H = Var(H) - -{Var(H) * ceg(A + E + S)} = = 8.158	simple effect of all predictors pseudo-*F* = 8.5, *p* = 0.001

In variation partitioning by RDA, on selected PCoA axes scores for H as response variables (Fig. S2 and Table S4), with A, E, and S as predictor groups, all simple effects were significant (p_{adj} < 0.005). The analyses of conditional terms, revealed significant unique effect of S (pseudo-$F = 7.6$, $p = 0.004$), while the unique effects of E (pseudo-$F = 2.6$, $p = 0.062$) and A (pseudo-F = 2.5, $p = 0.073$) were marginally significant ($p < 0.08$). The unadjusted explained variation was 79.6%.

Equations used for variation partitioning in this example and the canonical eigenvalues are given in Table S4. Using, in turn, the matrices as response data tables, the values from the following three figures emerge (Fig. S3 to S5).

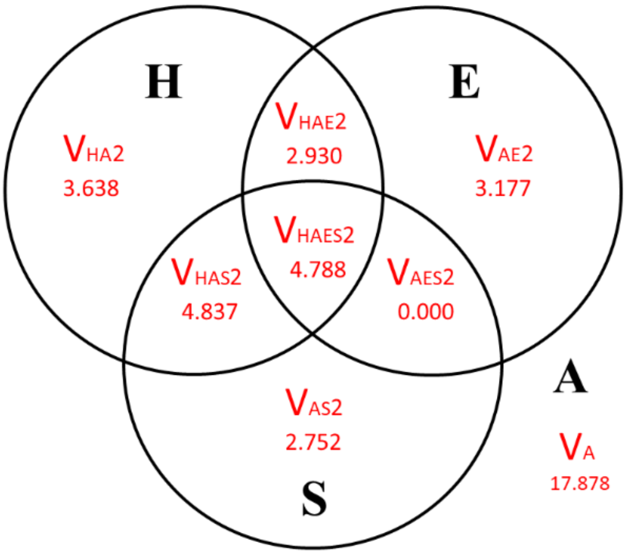

Fig. S3. Variation partitioning (Venn) diagram, showing the unique and shared effects of the predictors (H—natives, E—environment, and S—spatial eigenvectors) on A—the community of alien invasive species. Selected PCoA scores are used for each matrix. The variability explained by each combination of predictors (unique and shared) and the residual variation of the response matrix are given in red; the number in codes corresponds to the current analysis.

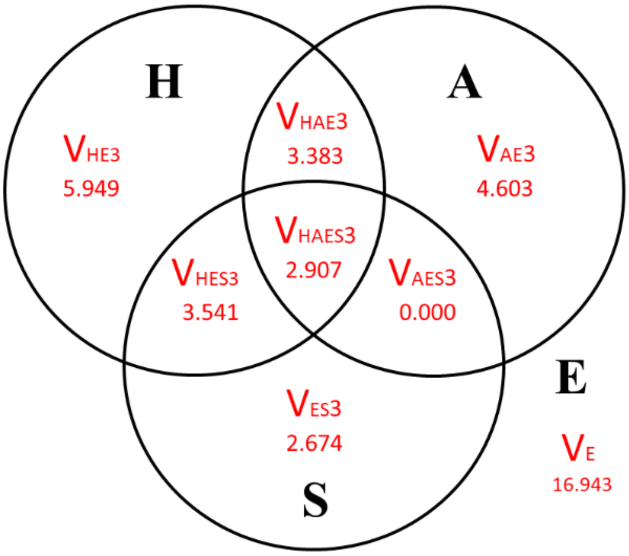

Fig. S4. Variation partitioning (Venn) diagram showing the unique and shared effects of the predictors (H—natives, A—alien invasive, and S—spatial eigenvectors) on the E—environmental matrix. Selected PCoA scores are used for each matrix. The values of variability explained by each combination of predictors (unique and shared) as well as the residual variation of the response matrix are given in red; the number in codes corresponds to the current analysis.

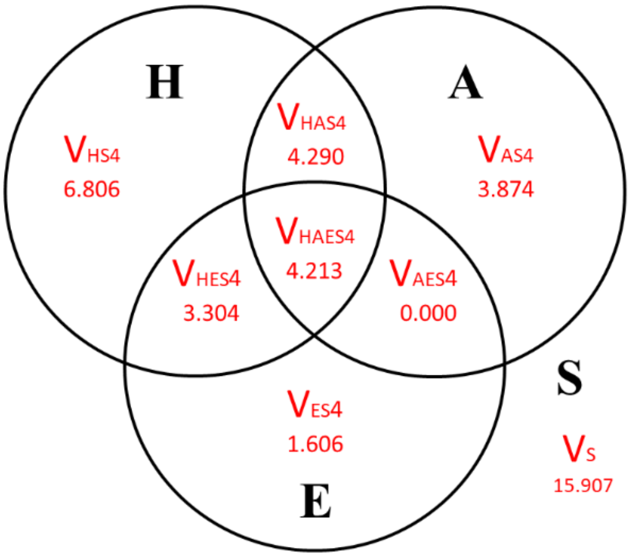

Fig. S5. Variation partitioning (Venn) diagram, showing the unique and shared effects of the predictors (H—natives, A—alien invasive, and E—environment) on S—spatial eigenvectors matrix. Selected PCoA scores are used for each matrix. The values of variability explained by each combination of predictors (unique and shared) as well as the residual variation of the response matrix are given in red; the number in codes corresponds to the current analysis.

Step 5. A model was built as a table, depicting all parts of variation partitioning, expressed as the value of explained variation and as percent, the values obtained in the former steps being summed (Table S5). The graphical representation is given in the main text (Fig. 7) and an alternative illustration in Fig. S6, these being newly introduced as CUVARP diagrams.

Table S5. Cumulative variation partitioning of all interactions between the four data sets (selected PCoA axes scores: H—natives, A—alien invasives, E—environment, S—space), each being used in turn as a response table. Values of the variation parts (upper values, upright) were summed up and expressed as percentages of the total variation (lower values, italicized). CV—cumulative variation; individual values of the part of explained variation correspond to the codes in Fig. S2 to S5 (sum of all variation in all tables is 160, representing 100%). Ø—residual (unexplained) variation.

	**Ø**	**E**	**ES**	**S**
**Ø**		$CV_{E} = V_{E} =$<br>= 16.943<br>*(10.59%)*	$CV_{ES} = V_{ES3}+V_{ES4} =$<br>= 4.280<br>*(2.68%)*	$CV_{S} = V_{S} =$<br>= 15.907<br>*(9.94%)*
**H**	$CV_{H} = V_{H} =$<br>= 8.158<br>*(5.10%)*	$CV_{HE} = V_{HE1}+V_{HE3}=$<br>= 9.225<br>*(5.77%)*	$CV_{HES} = V_{HES1} +$<br>$+ V_{HES3} + V_{HES4} =$<br>8.747<br>*(5.47%)*	$CV_{HS} =$<br>$V_{HS1}+V_{HS4}=$<br>= 16.402<br>*(10.25%)*
**HA**	$CV_{HA} =$<br>$V_{HA1}+V_{HA2}=$<br>= 6.759<br>*(4.22%)*	$CV_{HAE} = V_{HAE1} +$<br>$+ V_{HAE2} + V_{HAE3} =$<br>= 12.824<br>*(8.02%)*	$CV_{HAES} = V_{HAES1} +$<br>$+ V_{HAES2} + V_{HAES3} +$<br>$V_{HAES4} = 17.258$<br>*(10.78%)*	$CV_{HAS} = V_{HAS1} +$<br>$+ V_{HAS2} + V_{HAS4} =$<br>11.213<br>*(7.01%)*
**A**	$CV_{A} = V_{A} =$<br>= 17.878<br>*(11.17%)*	$CV_{AE} = V_{AE2}+V_{AE3}=$<br>= 7.780<br>*(4.86%)*	$CV_{AES} = V_{AES2} +$<br>$+ V_{AES3} + V_{AES4} =$<br>= 0.000<br>*(0.00%)*	$CV_{AS} =$<br>$V_{AS2}+V_{AS4}=$<br>= 6.626<br>*(4.14%)*

The percentual data from Table S5 are depicted in the CUVARP diagram in Fig. 7 (from the main text), and an alternative is a 3D display with cones in Fig. S6.

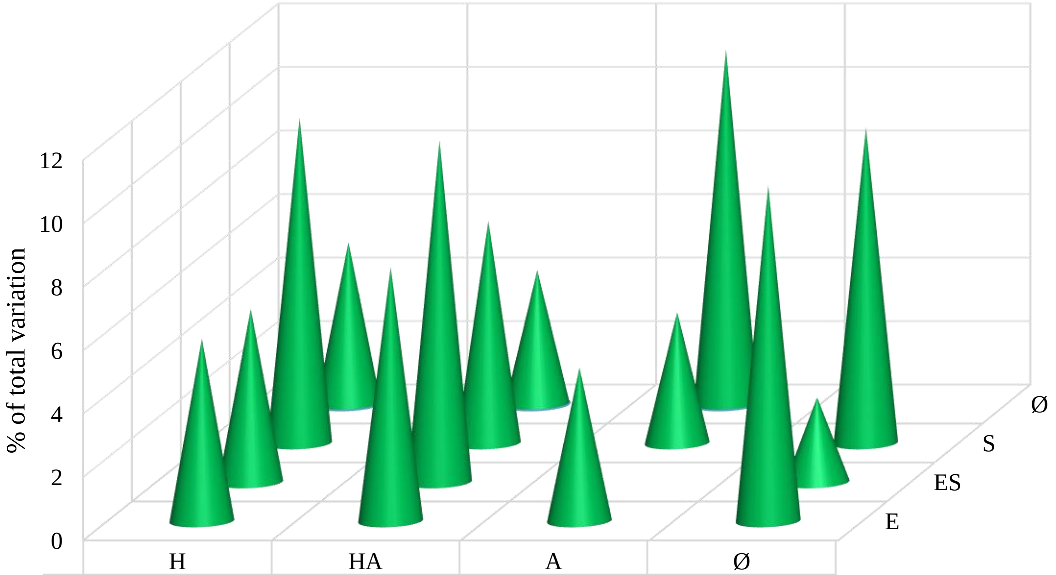

	H	HA	A	Ø
E	5.77	8.02	4.86	10.59
ES	5.47	10.78	0	2.68
S	10.25	7.01	4.14	9.94
Ø	5.1	4.22	11.17	

Fig. S6. Alternative CUVARP diagram (cumulative variation partitioning with multiple response and predictor matrices). All datasets are synthesized through selected PCoA axes scores of H—native species, A—alien invasives, E—environmental parameters, and S—space. Ø—unexplained (residual) variation. The percentage of variation (explained and residual) from the total variation in all matrices is depicted.

Methodological critical analysis, alternatives to the CUVARP algorithm and proposals for expanding the framework

Our research, which continues and adds to our previous work [7S], shows the importance of interdisciplinary analyses and approaches on any subject of major concern in ecology. While this statement is generally accepted, little is done to develop a sound methodological basis for interdisciplinary analysis of ecological systems and even less for the multi-matrix (multiple data tables) analyses of ecological data. We consider this needs to be added to the classical multivariate analyses paradigm. However, our algorithm [7S] included only one response matrix: the community or site-by-species table. We also ascertain that multiple communities (response matrices) can and should be analyzed and compared, and here we propose a method for this.

If matrices (e.g., the ecological niches' dissimilarities between species) [7S] are unavailable or not desired, our approach also enables the use of vectors (or variables) of synthetic measures such as species' responses or tolerances to different environmental factors. However, variables or vectors hold a single value for one species' response to a certain predictor, not accounting for shifts, alternate responses, and probabilities of individual or group responses. In contrast, our approach based on matrices (also developed in [7S]) allows for all these and many more, such as multi-trajectory dynamics and unstable balance, multiple statistical responses of populations and species, and distribution of preferred resources' use. In short, we stress that future community ecology should be based on the principle "matrices instead of variables (or descriptors, or vectors)." Putting together our approaches of multi-matrix analyses, the use of matrices instead of variables, and non-commutative

relationships between analyzed data tables describing life and environmental features, we show that all these reflect some basic principles of quantum physics. Thus, the future may offer a developing interest for some border- and intersection of sciences, such as "quantum ecology," or at least a "quantum physics principles-based ecological research."

The novel CUVARP analysis was performed on scores of the selected PCoA axes of the original data tables. Another potential variant is to perform these analyses using community weighted means (CWM) of natives and AIS in response to their functional traits. Trait-based ecology is trending: it is continuously developing, including assessing dimensionality, robustness, and structure of trait spaces [8S]. Reduced dimensionality by computing PCoA axes scores using the Gower distance for the trait matrix, followed by the use of the result in a forward selection procedure for selecting those axes scores that are significantly related to the response data table, might also be used in linking multiple matrices and exploring their relations. Multivariate variation partitioning methods continue to develop, as are their applications, types of data used, and terminology [9S, 10S, 11S, 12S, 13S, 14S, 15S]. They have recently been employed to address the problem of variation among species and sites, called the "internal structure" of the metacommunity, contributing to the development of a more cohesive metacommunity theory [16S]. Trait matrices in multidimensional community analyses were used in variation partitioning in double-constrained ordination analyses, leading to a new graphical display (called VADOC diagrams) and definition of "omni-spaces explanatory ecology of communities" [7S].

Almost all articles and research related to our approach concern communities as response data tables, while other matrices act as predictors. We have demonstrated the utility of the reverse approach by addressing the question of the explanatory power of communities on environment and space, the effect of AIS on natives combined with the symmetrical cumulative ambivalent effect of all components of the model. Asking questions about how communities predict the variability in environmental features might also inspire a future quest for how different communities interfere in their relationships with external factors. We assume that an amount of environmental and spatial variability can sustain a certain density of interactive communities. Thus, a diversity of habitats and external factors might be linked to communities' structural, functional, and niche-based diversity (sensu Sîrbu et al. [7S]).

Our algorithm might seem odd and difficult to comprehend because after applying PCoA, saving, and further using scores for predictors and species in new analyses, the results might seem far from the original data. There are several reasons for not applying the algorithm directly to the original data sets. Among these is the frequently encountered limitation of having many predictors in several data tables that will be linked, while the number of cases (samples, communities) is low. Many variables and a few cases turn the direct ordination method into an indirect (unconstrained) analysis. Then, there is the problem of having variables (especially predictors) of different types varying on various scales. While a standardization might work for quantitative variables, using in various analyses a combination of quantitative, ordinal, and factorial variables that serve either as predictors or responses or both leads to inadequate conditions for applying different canonical methods. Using the Gower distance (which can calculate distance on any group of variables) and turning the distance matrix using a PCoA into a rectangular data table with scores on the axes is a solution in many applications. When communities are of different species richness, a direct selection might lead to selecting only some species, usually a few; thus, the results and conclusions will not be drawn at the community level but will refer only to some particular taxa. If space coordinates are involved, applying db-MEM might show (as is our case) complex environmentally space-structured communities variation. In contrast, the direct use of coordinates or their trend-surface polynomials is fit for monotonic or smooth non-linear changes, unable to reveal other types of spatial heterogeneity patterns [1S]. If the data tables are square, meaning a set of distances (such as dissimilarities, niche overlap measures, genetic distances), applying a distance-based redundancy analysis (db-RDA) can be a viable alternative, this being already a canonical PCoA analysis. However, we acknowledge that there are also alternatives to our algorithm. In some cases, applying the algorithm directly to the original data sets can be done, e.g., if in every data table there are fewer predictors than cases, if

studies are focused not on all communities components but on some particular species that might turn out to be significantly related to the other variables, in homogenous data sets and others.

Expanding or alternatives to the CUVARP analysis and diagrams might also be done in other ways:

(1) In Table S5, the values from each cell might be expressed as average (e.g., arithmetic mean), the proportions being calculated on the sum of these statistics. The result might be termed as an AVARP diagram (A standing for average or mean values).

(2) Instead of PCoA axes scores of original matrices (counts of species), one might also wish to include functional traits, such as community weighted means or CWM, which are calculated on sites-by-species (counts or other ecological parameters) and species-by-functional traits data tables. These CWM are further expressed as PCoA axes scores (separately for native and AIS communities); the new matrices are subject to an interactive forward selection. The selected PCoA axes scores of natives are the new data table denoted "N" and the corresponding code for the alien species scores and data table is "I". The rest of the algorithm is applied accordingly. The result (table and diagram) might be termed as CWM-CUVARP (from communities weighted means-based cumulative variation partitioning with multiple response and predictor data tables), respectively as CWM-AVARP (if average values replace the cumulative variation parts). We are introducing and illustrating here the Community Weighted Means - Average Variation Partitioning with Multiple Response and Predictor Matrices Diagram on our data (Fig. S7).

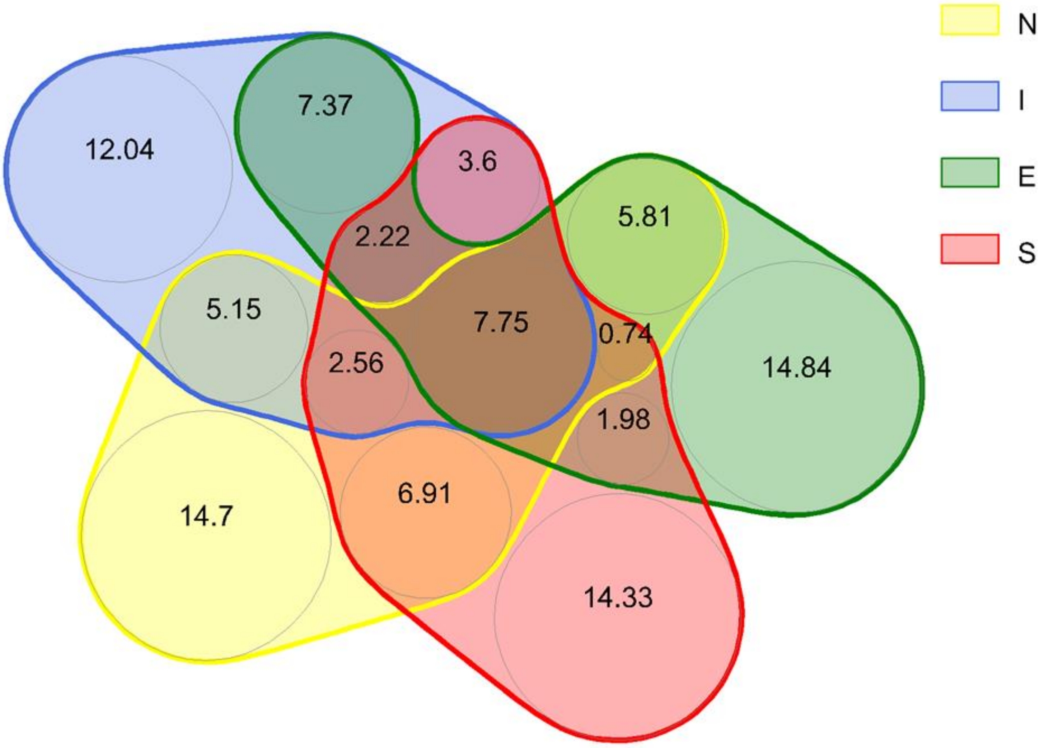

Fig. S7. CWM-AVARP (Community Weighted Means - Average Variation Partitioning with Multiple Response and Predictor Matrices) diagram. All datasets are synthesized through selected PCoA axes scores of communities weighted means (CWM, computed on site-by-species and species-by-functional traits data tables) of N—native species, I—alien invasives, E—environmental parameters, and S—space. No overlapping colored circles depict unexplained (residual) variation. The percentage of variation (explained and residual) from the total variation in all matrices is shown.

(3) In alternative analyses, it is possible to use, instead of the devoted canonical ordination methods (CCA, RDA), correlative methods that analyze relations between data tables, such as the Co-correspondence analysis (CoCA), the co-inertia analysis (CoIA), co-correlation analysis (CCorA), or the Procrustes rotation analysis (ProcA). Some of these (especially the CoCA and CoIA) have certain advantages, such as the possibility of proper use when the number of variables

(predictors included) is large compared to (or even surpasses) the number of cases. But there is a problem: CUVARP (and our formerly defined method of variation partitioning in dc-CA, namely the VADOC [7S]) are methods of variation partitioning, and among the requirements, they need to assess unique and shared effects, and to test at least some of these. This means the necessity of analyses done with covariates (partial canonical analyses). But, to the best of our knowledge, the methods described before (e.g., CoCA, CoIA) have not yet been developed to work with covariates for partialling out the effect of some variables. Thus, the use of such methods still needs to be developed.

(4) The necessity of linking multi-space datasets also leads to finding new methods for data visualization since the plain paper and computer screens do not support details and have already reached their limits. A first step will probably be developing 3D interactive graphics capabilities in future scientific journals. The readers will use some digital optic devices and see a virtual figure that can be rotated or magnified in real-time to understand its content and meaning better. The development of such new publishing methods is already necessary.

References used in the Supplementary Material

1S. Šmilauer, P. & Lepš, J. *Multivariate Analysis of Ecological Data using Canoco 5*. (Cambridge University Press, 2014).
2S. Ter Braak, C. J. F. & Šmilauer, P. *Canoco reference manual and user's guide: software for ordination (version 5.10)* (Microcomputer Power, 2018).
3S. Rao CR. Diversity and dissimilarity coefficients: A unified approach. *Theor. Popul. Biol.* 21, 24–43 (1982).
4S. Lepš J, de Bello F, Lavorel S & Berman S. Quantifying and interpreting functional diversity of natural communities: practical considerations matter. Preslia 78, 481–501 (2006).
5S. Legendre, P. & Legendre, L. *Numerical Ecology* (Elsevier, 2012).
6S. Ter Braak, C. J. F., Šmilauer, P. & Dray, S. Algorithms and biplots for double constrained correspondence analysis. *Environ. Ecol. Stat.* **25**, 171–197 (2018).
7S. Sîrbu, I., Benedek, A.M. & Sîrbu, M. Variation partitioning in double-constrained multivariate analyses: linking communities, environment, space, functional traits, and ecological niches. *Oecologia* **197**, 43–59 (2021).
8S. Mouillot, D. *et al*. The dimensionality and structure of species trait spaces. *Ecol. Lett.* **24**, 1988–2009 (2021).
9S. Peres-Neto, P. R. & Legendre, P. Estimating and controlling for spatial structure in the study of ecological communities. *Glob. Ecol. Biogeogr.* **19**, 174–184 (2010).
10S. Legendre, P., Borcard, D. & Roberts, D. W. Variation partitioning involving orthogonal spatial eigenfunction submodels. *Ecology*. **93**, 1234-1240 (2012).
11S. Fründ, J. Dissimilarity of species interaction networks: how to partition rewiring and species turnover components. *Ecosphere*. **12**, e03653. https://doi.org/10.1002/ecs2.3653 (2021).
12S. Perkin, J. S., Papraniku, I. F., Gibbs, W. K., Hoeinghaus, D. J. & Walker, D. M. Temporal trajectories in metacommunity structure: Insights from interdisciplinary research in intermittent streams. *Wiley Interdiscip. Rev. Water*. **8,** e1531. https://doi.org/10.1002/wat2.1531 (2021).
13S. Ricotta, C., Pavoine, S., Cerabolini, B. E. L. & Pillar, V. D. A new method for indicator species analysis in the framework of multivariate analysis of variance. *J. Veg. Sci*. **32**, e13013. https://doi.org/10.1111/jvs.13013 (2021).
14S. Willig, M. R. & Presley, S. J. Long-term trends in gastropod abundance and biodiversity: Disentangling effects of press versus pulse disturbances. *Glob. Ecol. Biogeogr.*, **31**, 247–265 (2022)
15S. Lai, J., Zou, Y., Zhang, J., Peres-Neto, P. R. Generalizing hierarchical and variation partitioning in multiple regression and canonical analyses using the rdacca.hp R package. *Methods Ecol. Evol.* Early view, https://doi.org/10.1111/2041-210X.13800 (2022).
16S. Liebold, M. A. *et al*. The internal structure of metacommunities. *Oikos*. 2022. https://doi.org/10.1111/oik.08618 (2022).
